# Efficacy and tolerability of baclofen in a U.S. community population with alcohol use disorder: a dose-response, randomized, controlled trial

**DOI:** 10.1038/s41386-021-01055-w

**Published:** 2021-06-21

**Authors:** James C. Garbutt, Alexei B. Kampov-Polevoy, Cort Pedersen, Melissa Stansbury, Robyn Jordan, Laura Willing, Robert J. Gallop

**Affiliations:** 1 Department of Psychiatry, University of North Carolina at Chapel Hill, Chapel Hill, NC USA; 2 Bowles Center for Alcohol Studies, University of North Carolina at Chapel Hill, Chapel Hill, NC USA; 3 Department of Mathematics, Applied Statistics Program, West Chester University, West Chester, PA USA

**Keywords:** Drug development, Psychiatric disorders

## Abstract

Identification of new medications for alcohol use disorder (AUD) is important for improving treatment options. Baclofen, a GABA_B_ agonist, has been identified as a potential pharmacotherapy for AUD. In a 16-week double-blind, randomized, placebo-controlled trial, we investigated 30 and 90 mg/day of baclofen compared to placebo and examined effects of dose, sex, and level of pretreatment drinking. One hundred and twenty participants with DSM-IV alcohol dependence (age 46.1 (sd = 10.1) years, 51.7% male) were randomized after exclusion for unstable medical/psychiatric illness and/or dependence on drugs other than nicotine. Seventy-three participants completed the trial. A main effect of baclofen was found [%HDD (F(2,112) = 4.16, *p* = 0.018, *d* = 0.51 95%CI (0.06–0.95), 13.6 fewer HDD) and %ABST (F(2,112) = 3.68, *p* = 0.028, *d* = 0.49 95%CI (0.04–0.93), 12.9 more abstinent days)] and was driven by the 90 mg/day dose. A sex × dose interaction effect was present for both %HDD (F(2,110) = 5.48, *p* = 0.005) and %ABST (F(2,110) = 3.19, *p* = 0.045). Men showed a marginally positive effect for 90 mg/day compared to PBO (%HDD t(110) = 1.88, *p* = 0.063, *d* = 0.36 95%CI (−0.09–0.80), 15.8 fewer HDD days; %ABST t(110) = 1.68 (*p* = 0.096, *d* = 0.32 95%CI (−0.12–0.76), 15.7 more ABST)) with no effect for 30 mg/day. Women showed a positive effect for 30 mg/day (%HDD, t(110) = 3.19, *p* = 0.002, *d* = 0.61 95%CI (0.16–1.05), 26.3 fewer HDD days; %ABST t(110) = 2.73, *p* = 0.007, *d* = 0.52 95%CI (0.07–0.96), 25.4 more ABST days) with marginal effects for 90 mg/day on %ABST (*p* = 0.06) with drop-outs/dose reduction from sedative side-effects of 59% in women at 90 mg/day compared to 5% for men. These findings support the hypothesis that baclofen has efficacy in AUD and suggest that dose and sex be further explored as potential moderators of baclofen response and tolerability.

## Introduction

Alcohol use disorder (AUD) are some of the most common and destructive medical disorders in the United States and the world yet, in the US, <20% of individuals with an AUD seek treatment [1]. Furthermore, the use of FDA-approved medications for AUD is low [2] despite evidence of their efficacy [3]. Nevertheless, medications are emerging as an important component of treatment as it becomes increasingly clear that the pathophysiology of AUD includes a strong genetic/neurobiological basis amenable to pharmacological treatment [4].

Currently, there are four FDA-approved medications for AUD (disulfiram, oral naltrexone, long-acting intramuscular naltrexone, acamprosate) and a number of non-FDA-approved medications have demonstrated efficacy in randomized controlled trials, e.g. topiramate [3], gabapentin [5], and nalmefene has been approved in Europe and Japan. An issue with these medications is that their overall effect size in heterogeneous populations is low [6]—a factor in their low utilization [2]. Accordingly, efforts to identify new medications that may target individuals unresponsive to existing medications or provide other advantages, e.g. safety with liver disease, are ongoing.

Baclofen, a GABA_B_ receptor agonist, is a relatively new medication for AUD that has demonstrated efficacy in some but not all clinical trials (for meta-analyses, see [7–9]), with some indications that higher doses of baclofen (>60 mg/day) are less tolerated and less effective than lower doses [7] even though open-label and case report studies have advocated for enhanced efficacy of baclofen with doses up to 300 mg/day, e.g., see de Beaurepaire [10]. Pierce et al. [7] found some evidence that higher levels of pretreatment alcohol consumption were associated with a stronger response to baclofen. Baclofen has also attracted interest in the medical community as it is the only medication, to date, that has shown efficacy for reducing drinking safely in patients with clinically relevant alcohol-associated liver disease [11, 12]. For a recent consensus statement on the use of baclofen for AUD see Agabio et al. [13].

Within the US, RCTs of baclofen for AUDs have shown no superiority of baclofen over placebo in a community population (*n* = 80, baclofen 30 mg/day) though anxiety improved [14] or in a hepatits C population in veterans (*n* = 180, baclofen 30 mg/day) [15] whereas one trial [16] found some positive effects of 80 mg/day of baclofen in a sample of comorbid nicotine/alcohol-dependent individuals (*n* = 30). However, given the ongoing, albeit mixed, evidence that baclofen has value for AUD we conducted an RCT in a U.S. community AUD population comparing 90 mg/day to 30 mg/day of baclofen and to placebo and stratifying by sex and by the level of pretreatment drinking to test the hypothesis that baclofen was superior to placebo in reducing drinking behavior and that sex and level of pretreatment drinking may moderate response.

## Participants and methods

### Study design and oversight

The study was a three-arm, randomized, double-blind, placebo-controlled trial comparing placebo to 30 mg/day and to 90 mg/day of baclofen in a 1:1:1 randomized block assignment with block sizes of 6 (SAS software), stratified by sex and by pre-randomization level of alcohol consumption (high consumption defined as ≥14 standard drinks/drinking day for men and ≥10 standard drinks/drinking day for women (a standard drink contains ~14 g ethanol)). The study was conducted in an outpatient setting and approved by the Committee on the Protection of the Rights of Human Subjects, School of Medicine, University of North Carolina at Chapel Hill. All participants provided written informed consent prior to entry. The full trial protocol and statistical analysis plan are provided in Supplement 1. Participants were recruited from the community via advertising and were screened initially by phone followed by in-person interview and exam.

### Participant population

**Fig. 1:**
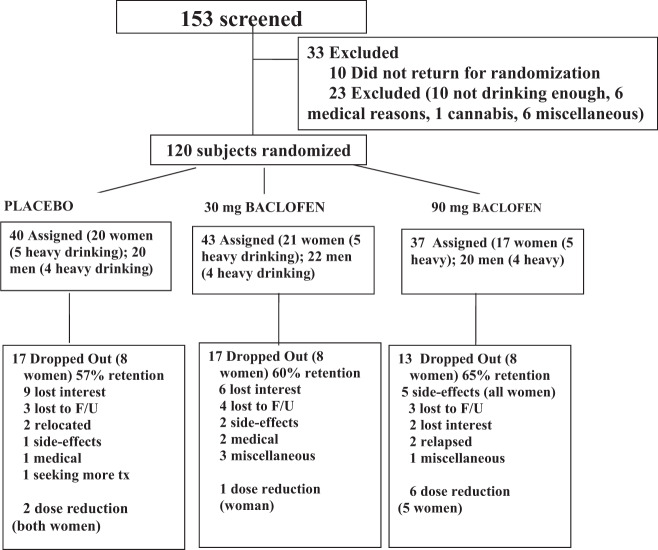
Consort diagram.

Four hundred and thirty individuals were screened by phone between December, 2013 and June, 2017 (15 excluded for drug use disorder) to yield 153 participants who were screened in-person to yield 120 randomized participants (see Fig. 1, CONSORT).

Participants needed to be between the ages of 21–65 years, to have a diagnosis of DSM-IV alcohol dependence [17], to have a reported drinking pattern of more than 14 standard drinks (women) or 21 drinks (men) per week including at least 2 heavy drinking days/week on average (men ≥ 5 standard drinks/day; women ≥4 standard drinks/day) during a consecutive 30-day period within the 90 days prior to screening based on Time-Line FollowBack (TLFB) [18] interview and to express an interest in significantly reducing or stopping alcohol use.

Individuals were excluded if they had a significant medical illness, e.g. diabetes mellitus, a significant psychiatric illness, e.g., bipolar disorder or were taking psychotropic medication with the exception of stable doses of antidepressants for at least one month, or had evidence of another substance use disorder with the exception of nicotine dependence or cannabis abuse (Supplement 1).

### Study procedures

At in-person screening, subjects signed informed consent and received a breath alcohol test which had to be 0.0 gms/dl in order to proceed with the screening visit. Subjects were medically evaluated including a liver panel, CBC, chemistry panel, serum pregnancy test, and urinalysis. A urine toxicology screen was completed. Alcohol use in the 90 days prior to screening was assessed by the TLFB, a Penn Alcohol Craving Scale (PACS) [19] and a Spielberger State-Trait Anxiety Inventory (STAI) [20], and a Mini International Neuropsychiatric Interview [21] to determine psychiatric diagnoses.

Participants who met criteria were randomized to either placebo, 30 mg/day baclofen or 90 mg/day baclofen using randomization blocks provided by Dr. Gallop [SAS Institute Inc. (2015). SAS® 9.4 Language Reference: Concepts, 5th Edition, Cary, NC: SAS] Institute Inc to the UNC Investigational Drug Service stratified by sex and level of drinking. Blinded baclofen/placebo was provided in blister packs to the research assistant, self-administered and titrated to 10 mg tid over 4 days or 30 mg tid over 12 days, and down-titrated from 90 to 0 mg from days 95 to 107 or from 30 to 0 mg from days 104 to 107 with terminal dose of 10 mg/day in both groups. Sobriety was not a requirement for randomization.

Participants were seen weekly for 4 weeks then biweekly until week 16 with TLFB, PACS, and STAI assessed and Medical Management (MM) [22], a 10–15 min menu-driven session focused on inquiry of side-effects, medication compliance, and progress toward drinking goal, provided at each visit by trained clinicians.

An 8 h urine sample (12:00 am to 8:00 am) was collected at week 4 to assess level of baclofen and frozen at −70 °C until assayed several months later, see Supplement 1 for assay method. AST, ALT, bilirubin, GGT, creatinine, and glucose were assessed at weeks 4, 8, 12, and 16. A %carbohydrate-deficient transferrin (CDT) was drawn at screening visit and at end of study, frozen at −70 °C and assayed in batches at the Medical University of South Carolina. A CDT/transferrin ratio ≥1.7 is considered evidence of heavy drinking in the past several months.

### Study end points

The two primary end points were % heavy drinking days (HDD) and % abstinent days (ABST) over the active treatment period as assessed by TLFB. For this intention-to-treat analysis all subjects were included in the analysis once randomized with the exception of two subjects who failed to provide any post-randomization data. The effect of baclofen dose, sex, and level of pre-randomization drinking on response to baclofen were a priori considerations given the evidence these may be moderators for efficacy or tolerability [7–10]. In addition, the effect of baclofen on state anxiety was a preplanned analysis. Secondary analyses included changes in PACS, responder analysis defined as no heavy drinking in final 8 weeks of trial [23], and changes in CDT/GGT.

### Statistical analysis

Reported baseline values are observed means with standard deviations for continuous measures and percentages for categorical responses. Baseline differences in demographic variables and level of baseline drinking between placebo, 30 mg/day baclofen, and 90 mg/day of baclofen were investigated using analysis of variance (ANOVA) models for continuous variables and Chi-square tests for categorical variables. Analytic results are given as model-based estimates with standard errors.

TLFB provides weekly scores of abstinence and heavy drinking for each subject during the 16 weeks of medication, resulting multiple observations within-subject. When the within-subject correlation is properly incorporated, the statistical framework results in an increase of statistical power over methods that compare groups cross-sectionally [24]. We implemented a general mixed model analysis of variance (MMANOVA) framework which models the means per group over time and the covariance between the repeated measures [25]. The terms in the MMANOVA model included treatment, gender, week, and patient’s pretreatment drinking status (heavy or moderate) with three aims: (a) assessment of the treatment effect, (b) assessment of the dependency of the treatment effect on gender, and (c) assessment of the dependency of the treatment effect on the pretreatment drinking status. The first is assessed through the main effects of intervention which represents the on-average difference over the longitudinal period. The second and third are assessed through two-way interaction of treatment within gender and pretreatment drinking status, separately. A treatment by week interaction and the three-way interaction of treatment within gender and pretreatment drinking status by week assess whether the treatment effect and the moderation effects vary differentially over the weeks of treatment. If non-significant, the two-way and three-way interactions with week are removed from the model.

Sensitivity analyses of the intervention effect are based on a recommended definition of clinical response [23]—no HDD over the last 8 weeks. Logistic regression analyses are used to accommodate these measures. For clinical significance, number needed to treat (NNT) is provided.

Both craving measured through the PACS and anxiety measured through the STAI are acquired repeatedly over the intervention period and analyzed using MMANOVA.

With repeated assessments, missing data/attrition is inevitable, but the key thing is that the specified contrasts are not affected due to the presence/absence of data. We used pattern-mixture models to assess if there is bias due to drop out or missing data [26]. As a sensitivity analysis of the primary outcome models, missing data are multiply imputed using chained-equations. Comparison of observed-data results and imputed-data results are made. Results based on the observed-data models are reported if results are consistent.

The study was designed to have a sample of 120 which yielded over 80% power to detect a difference of at least 13% in %HD/%ABS rates between BAC and PBO, while accounting for up to 16.2% attrition. The study had 80.2% power to detect moderation, categorized by a large effect for BAC compared to PBO (*d* = 1.2) observed in one level of the moderator and no difference (*d* = 0) in the other, where the large effect corresponds to a difference of 28% in %HD/%ABST rates between BAC and PBO.

All modeling approaches allow for the inclusion of covariates. We included any measures differing at baseline or important potentially confounding measures (i.e., gender, pretreatment drinking status) in our models. All analyses used SAS9.4. Statistical significance was set at *p* < 0.05 (two-tailed) for all tests. With respect to the dual outcomes as well as the multiple analyses (moderation models) as a sensitivity analysis to potential inflated type I error, we implemented the false discovery rate approach adjusting for the number of tests within each outcome (ABST & HDD).

## Results

### Subject recruitment and characteristics

Figure 1 shows the CONSORT diagram including retention. Demographic characteristics are shown in Table 1. Secondary analyses with these variables as covariates, including antidepressants, yielded the same pattern of results as those without the covariates.

**Table 1:** Baseline demographics and clinical measures, mean ± SD or % (*n* = *X*).

Measure	30 mg/day (*n* = 43)	90 mg/day (*n* = 37)	Placebo (*n* = 40)	Overall (*n* = 120)	Statistical significance
Proportion abstinence pre-90	0.17 (0.19)	0.18 (0.20)	0.13 (0.17)	0.16 (0.19)	0.52
Proportion heavy drinking pre-90	0.73 (0.23)	0.70 (0.26)	0.79 (0.21)	0.74 (0.23)	0.26
Mean drinks per drinking day pre-90	10.65 (7.68)	9.98 (4.20)	9.31 (3.90)	10.00 (5.61)	0.60
Abstinent day prior to randomization	14.0% (6)	18.9% (7)	15.0% (6)	15.8% (16)	0.82
Antidepressants	11.6% (5)	27.0% (10)	40.0% (16)	25.8% (31)	0.013
Antihypertensives	14.0% (6)	21.6% (8)	20.0% (8)	18.3% (22)	0.64
THC positive	14.0% (6)	13.5% (5)	22.5% (9)	16.7% (20)	0.48
CIWA	1.60 (2.16)	1.22 (1.57)	1.13 (1.38)	1.35 (1.75)	0.30
PACS total score	15.53 (7.18)	15.27 (6.13)	14.49 (6.77)	15.11 (6.69)	0.77
STA-anxiety	36.28 (12.41)	35.65 (9.67)	35.03 (10.46)	35.67 (10.90)	0.87
%Carbohydrate-deficient transferrin (CDT)	1.72 (0.63)	2.22 (1.46)	2.31 (1.95)	2.07 (1.46)	0.16
CDT ≥ 1.7 U/L	50.0% (20/40)	45.7% (16/35)	55.3% (21/38)	50.4% (57/113)	0.72
% Males	51.2% (22)	54.1% (20)	50.0% (22)	51.7% (62)	0.94
% Heavy use	23.3% (10)	24.3% (9)	22.5% (9)	23.3% (28/120)	0.98
%Hispanic	2.3% (1)	2.7% (1)	0% (0)	1.7% (2)	0.76
% White	86.1% (37)	81.1% (30)	90.0% (36)	85.8% (103)	0.61
%Employed	76.4% (33)	78.4% (29)	77.5% (31)	77.5% (93)	0.98
% Goal of total abstinence	55.8% (24)	35.1% (13)	40.0% (16)	44.2% (53)	0.14
% Any tobacco use	27.9% (12)	29.7% (11)	35.0% (14)	30.8% (37)	0.77
% Married	46.5% (20)	46.0% (17)	40.0% (16)	44.2% (53)	0.20
Education	15.9 (2.4)	16.0 (2.4)	14.8 (2.4)	15.6 (2.4)	0.047
Age	46.1 (10.4)	45.0 (10.4)	47.1 (9.6)	46.1 (10.1)	0.67

### Compliance

Returned pill counts revealed compliance rates of 96.3%, 98.1%, and 97.2% for PBO, 30 mg, and 90 mg, respectively.

Urine baclofen levels were used to examine for differences across groups and sex. Individuals assigned to 90 mg/day have higher levels (16.4 ± 6.9 μg/ml, *n* = 21) than those assigned to 30 mg/day (7.5 ± 3.3 μg/ml, *n* = 29) (t(46) = 5.95, *p* < 0.0001). Women and men on 90 mg/day have similar baclofen levels, 14.2 ± 8.1 μg/ml vs 18.5 ± 5.1 μg/ml, respectively, as well as on 30 mg/day, 7.3 ± 4.0 μg/ml vs 7.6 ± 2.5 μg/ml, respectively.

### Primary outcomes

#### Percent heavy drinking days

**Fig. 2:**
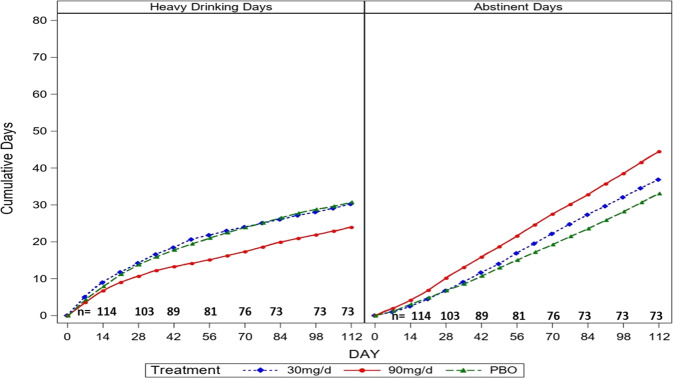
Cumulative days of heavy drinking and abstinent days over 16 weeks of treatment.

There is a significant baclofen effect to reduce heavy drinking days (F(2,112) = 4.16, *p* = 0.018, *d* = 0.51 95%CI (0.06–0.95), 13.6 fewer HDD) (see Fig. 2). This effect is driven by the 90 mg/day group (28% heavy drinking days) which demonstrates superiority to both PBO (39% heavy drinking days, *p* = 0.044, *d* = 0.39 95%CI (0.00–0.77), 11.7 fewer HDD) and 30 mg/day (42% heavy drinking days, *p* = 0.006, *d* = 0.53 95%CI (0.08–0.97), 15.6 fewer HDD). The baclofen effect was not moderated by treatment week (F(30,113) = 1.13, *p* = 0.28).

**Fig. 3:**
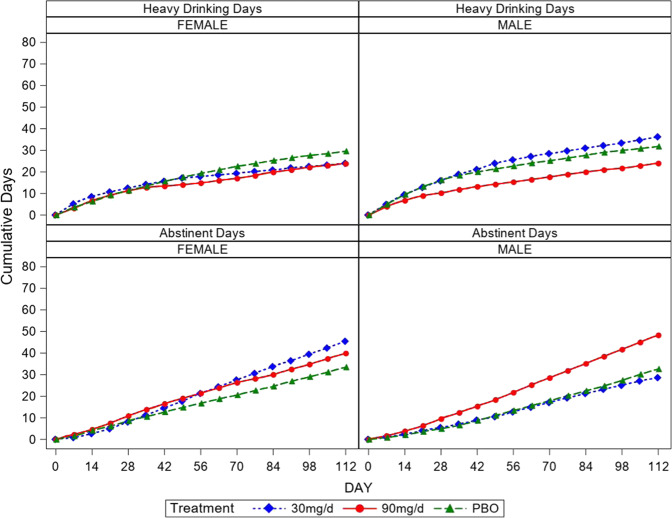
Cumulative days of heavy drinking and abstinent for men and women over 16 weeks of treatment.

Assessing the moderation effect for gender yields a significant interaction (F(2,110) = 5.48, *p* = 0.005) (see Fig. 3). Within women, 30 mg/day (22% HDD) is superior to PBO (45% HDD) (*p* = 0.002, *d* = 0.61 95%CI (0.16–1.05), 26.3 fewer HDD), whereas 90 mg (37% HDD) (*p* = 0.33, *d* = 0.19 95%CI (−0.25–0.63), 8.8 fewer HDD) is not. For men, 90 mg/day (26% HDD) is marginally superior to PBO (40% HDD) (*p* = 0.063, *d* = 0.36 95%CI (−0.09–0.80), 15.8 fewer HDD) whereas 30 mg/day (43% HDD) is not different from PBO (40%HDD) (*p* = 0.69). No moderation effect of pretreatment heavy drinking is seen (F(2,111) = 0.09, *p* = 0.92). No moderation effect of tobacco use is seen (F(2,111) = 0.70, *p* = 0.50).

#### Percent days abstinent

There is a significant baclofen effect to increase abstinent days (F(2,112) = 3.68, *p* = 0.028, *d* = 0.49 95%CI (0.04–0.93), 12.9 more abstinent days) (see Fig. 2). This effect is driven by the 90 mg/day group (59% ABST) which demonstrates superiority to both PBO (47% ABST) (*p* = 0.015, *d* = 0.47 95%CI (0.02–0.91), 13.8 more abstinent days) and 30 mg/day (48% ABST) (*p* = 0.027, *d* = 0.43 95%CI (0.01–0.90), 12.3 more abstinent days). The baclofen effect was not moderated by treatment week (F(30,113) = 0.89, *p* = 0.64). Assessing the moderation effect for gender yields a significant interaction (F(2,110) = 3.19, *p* = 0.045) (see Fig. 3). Within women, 30 mg/day (61% ABST) is superior to PBO (38% ABST) (t(110) = 2.73, *p* = 0.007, *d* = 0.52 95%CI (0.07–0.96), 25.4 more ABST) and 90 mg/day (55%ABST) is marginally superior to PBO (t(110) = 1.88, *p* = 0.063, *d* = 0.36 95%CI (−0.09–0.80), 18.9 more ABST). Within men, the 90 mg/day group (61% ABST) demonstrates marginal superiority to PBO (47% ABST) (t(110) = 1.68, *p* = 0.096, *d* = 0.32 95%CI (−0.12–0.76), 15.7 more ABST) whereas no difference between 30 mg/day (43%ABST) and PBO is seen (t(110) = 0.51, *p* = 0.61). No moderation effect of pretreatment heavy drinking was seen (F(2,111) = 0.92, *p* = 0.40). No moderation effect of tobacco use is seen (F(2,111) = 0.22, *p* = 0.80).

### Secondary outcomes

#### Responder analysis

There is a marginally significant overall intervention effect in the percent free of HDD during the last 8 weeks (F(2,113) = 2.43, *p* = 0.092). There is a significant effect for 90 mg/day (18% free of HDD) compared to PBO (5% free HDD) (t(120) = 2.12, *p* = 0.036, *d* = 0.39 95%CI (0.00–0.77), NNT = 7.7 95%CI (3.7–99.0) but no significant effect for 30 mg/day (9% free HDD, NNT = 11.1 95%CI (4.2–99.9).

Complete abstinence rates during the last 8 weeks are 3%, 4%, and 8% for PBO, 30 mg/day, and 90 mg/day, respectively (F(2,113) = 0.84, *p* = 0.43).

#### CDT

Fitting a logistic regression model for CDT at or above the 1.7 threshold there is a non-significant overall intervention effect (Chi(2) = 1.35, *p* = 0.51) with endpoint prevalance rates of 38.9%, 40.0%, and 47.1% for elevated CDT for PBO, 30 mg/day, and 90 mg/day, respectively.

#### GGT

Fitting a generalized linear mixed-effects model for the GGT outcome with normal upper limits defined as >109 U/L for men and >48 U/L for women based on the available data from week 8 through the end of treatment, there is a non-significant overall intervention effect (Chi(2) = 1.48, *p* = 0.23) while adjusting for screening GGT levels. Prevalence rates are 17.1%, 13.5%, and 6.1% for elevated GGT at end of treatment for PBO, 30 mg/day, and 90 mg/day, respectively, with no significant pairwise comparisons.

### Craving for alcohol

There is a non-significant intervention effect on craving (F(2,115) = 0.84, *p* = 0.44). All pairwise comparisons are non-significant (*p* > 0.20).

### Anxiety

There is a non-significant baclofen effect on anxiety (F(2,115) = 0.65, *p* = 0.52). A moderation effect is seen in subjects with high anxiety (≥40 STAI) at screening (F2,112) = 3.11, *p* = 0.048). Subjects with high anxiety prior to treatment demonstrate a positive effect of baclofen (F(2,113) = 4.83, *p* = 0.01) with on-average end-of-trial anxiety estimates of 43.4 for PBO, 43.1 for 30 mg/day, and 38.6 for 90 mg/day with a significant difference between 90 mg/day and PBO (*p* = 0.007) and 90 mg/day with 30 mg/day (*p* = 0.015).

### Tolerability

#### Adverse events

There was one serious adverse event, a woman on 90 mg/day who briefly fell asleep while driving but did not have an accident. Adverse events occurring in at least 5% of subjects within a treatment arm are shown in Table 2 by sex. The great majority of these were mild and not troublesome to the participant. Overall, we see significantly higher prevalence for tired/fatigue within women compared to men (*p* = 0.024). We see marginally higher prevalence for women compared to men for somnolence/sedation/drowsiness (*p* = 0.099) and headaches (*p* = 0.068). We have a significantly higher prevalence for at least one AE for women compared to men (*p* = 0.008).

**Table 2:** Adverse events.

Adverse event	Overall			Men			Women		
	TX			TX			TX		
	30 mg (*n* = 43)	90 mg (*n* = 37)	PBO (*n* = 40)	30 mg (*n* = 22)	90 mg (*n* = 20)	PBO (*n* = 20)	30 mg (*n* = 21)	90 mg (*n* = 17)	PBO (*n* = 20)
Sedation	60.5%^a^	54.1%^ab^	35.0%^b^	59.1%^a^	45.0%^ab^	20.0%^b^	61.9%	64.7%	50.0%
Dizziness	30.2%	18.9%	15.0%	22.7%	10.0%	15.0%	38.1%	29.4%	15.0%
Itchiness/rash	16.3%^a^	0%^**b**^	20.0%^a^	13.6%	0%	10.0%	19.1%^ab^	0%^a^	30.0%^b^
Tired/fatigue	0%	5.4%	7.5%	0%	0%	0%	0%	11.8%	15.0%
Nausea	16.3%	8.1%	10.0%	18.2%	5.0%	5.0%	14.3%	11.8%	15.0%
Constipation	9.3%	2.7%	7.5%	13.6%	0%	5.0%	4.8%	5.9%	10.0%
Headache	9.3%	10.8%	10.0%	9.1%	0%	5.0%	9.5%	23.5%	15.0%
At least one AE	76.7%	62.2%	60.0%	72.7%^a^	50.0%^ab^	35.0%^b^	80.9%	76.5%	85.0%

Note: percentages followed by the same superscripts are not pairwise statistically significantly different (*p* ≥ 0.05). No superscripts indicate no significant pairwise difference (*p* ≥ 0.05).

Drop-outs related to adverse events are shown in the CONSORT diagram. Drop-outs from side-effects are much more common in the 90 mg dose arm and are all women. Furthermore, drop-outs/reduction in dose from sedative side-effects affected 59% of women and 5% of men in the 90 mg arm compared to 15% and 0% in placebo and 14% and 5% in 30 mg arms, respectively, corresponding to a significant moderation effect due to gender by treatment (F(2,114) = 5.70, *p* = 0.004).

## Discussion

The current trial finds evidence that baclofen is superior to placebo in reducing heavy drinking days and increasing abstinent days in individuals with DSM-IV alcohol dependence. A reduction of 13.6 heavy drinking days and an increase of 12.9 abstinent days over 16 weeks compared to placebo is clinically meaningful and an indication of the progress that can build on patients’ motivation to reduce or stop drinking [27, 28]. The baclofen effect is most notable in subjects receiving 90 mg/day of baclofen with effect sizes of *d* = 0.39 95%CI (0.00–0.77) (reduction in heavy drinking days) and *d* = 0.47 95%CI (0.02–0.91) (increases in abstinent days) compared to PBO. These are medium effect sizes and higher than the overall small effect sizes reported for naltrexone and acamprosate [6]. However, meta-analyses of baclofen have shown mixed results [7–9] so the current results from a single-site trial cannot be taken as an indicator of overall baclofen effect size in alcohol use disorders. Nevertheless, the data indicate that baclofen may have value in treating patients with alcohol use disorders.

Baclofen was initially shown to have efficacy in alcohol use disorders in a small trial in Italy [26]. Since then, ~1500 subjects have been studied in placebo-controlled clinical trials primarily in Europe with several meta-analyses published [7–9]. Two main points emerge from this work. First, is that published trials have been mixed with some reporting no benefit to baclofen and others showing efficacy on total abstinence and time to lapse [7]. These mixed findings are not unusual in the alcohol use disorder field, e.g., two large U.S. trials [29, 30] failed to show efficacy of acamprosate though the FDA-approved acamprosate based on European trial data. Thus, the current trial adds to the body of work indicating that baclofen has value for AUD with questions remaining as to what are the effect sizes for different outcomes in broad populations with AUD and whether there are moderators of response that could help with prescribing guidance. With regards to moderators, we find evidence for an effect of sex but not for prior level of alcohol consumption on response to baclofen. Men did well on 90 mg/day, moderate effect sizes with marginal statistical superiority, but showed no effect at 30 mg/day. Women showed a significant effect at 30 mg/day with marginal effects at 90 mg/day.

It is of interest that women who received 90 mg/day baclofen had poor tolerability with 58% dropping out or reducing dose primarily related to sedation/drowsiness. This is consistent with reports that women have more adverse effects from baclofen than men [31]. However, in the current trial, tolerability in men was quite good even at 90 mg/day. While limited, our urine baclofen data did not indicate that women had higher levels of baclofen than men suggesting that the observed increase in CNS effects of baclofen in women may be a pharmacodynamic effect. Whether a slower upward titration of baclofen, e.g. increases of 5 mg/day every week or so, in women would improve tolerability merits consideration (Stafford, personal communication).

One small trial [32] noted a positive effect of baclofen 80 mg/day on days abstinent from alcohol and tobacco in individuals with comorbid AUD and tobacco dependence. We did examine tobacco as a potential moderator but did not find an effect though the power was very low.

There is evidence, post hoc analysis, that 90 mg of baclofen reduces anxiety in subjects with high, pre-trial anxiety in keeping with some prior trials [14]. If confirmed, baclofen could represent a therapeutic option in patients with AUD and anxiety, a common problem.

The current findings should also be considered with regards to baclofen’s reported safety and efficacy in patients with clinically relevant alcohol-associated liver disease [11, 12] and the preliminary evidence that baclofen may reduce alcohol withdrawal symptoms [33, 34]. A medication with safety in liver disease and one that may positively impact alcohol withdrawal symptoms, even if not used as a primary withdrawal agent where benzodiazepines remain the treatment of choice because of their clear anti-seizure and anti-delirium tremens actions [35], along with improvements in drinking outcomes merits investigation in populations with severe alcohol use disorder in hospital. Such an intervention could appeal to hospitalists [36]. Finally, alternative approaches to activating the GABA_B_ receptor such as with positive allosteric receptor modulators show promise as they have been shown to reduce alcohol intake in preclinical models with minimal sedation [37–39].

Limitations to the trial include a retention rate of about 60% across treatment arms, though similar rates are noted in recent baclofen trials [12, 40]. The population for the trial was drawn from a non-treatment seeking general community and therefore has less generalizability to more severely ill populations. Furthermore, extrapolation to African-American or other ethnic groups is not possible given the predominance of Caucasians in the trial. In addition, baclofen doses above 90 mg/day were not tested. Of the available weekly usage measures, 29.1% were missing due to attrition/withdrawal. Pattern-mixture models showed no significant informative effect for either intervention effect or moderation effect wth gender (*p* > 0.22). Results from the multiple imputation models remained consistent with the observed-data models (*p* < 0.05).

In summary, the current trial finds evidence for efficacy of baclofen to reduce heavy drinking and increase abstinent days in individuals with alcohol use disorders with evidence that 90 mg/day has greater efficacy overall though with reduced tolerability in women. Sex emerges as moderator of response with men benefiting from 90 mg of baclofen/day but not from 30 mg/day whereas women show benefit from 30 mg/day of baclofen, marginal benefit from 90 mg/day, and increased intolerability at 90 mg/day.

## Funding and disclosure

This work was supported by National Institute of Alcohol Abuse and Alcoholism Grant, R01AA020824. The Sponsor provided support for personnel, medication, laboratory assessments, and subject recruitment and payments. None of the authors have any conflicts of interest to disclose.

## Supplementary information

Protocol

## References

[1] Grant BF, Goldstein RB, Saha TD, Chou SP, Jung J, Zhang H (2015). Epidemiology of DSM-5 alcohol use disorder results from the national epidemiologic survey on alcohol and related conditions III. JAMA Psychiatry.

[2] Mark TL, Kranzler HR, Song X (2003). Understanding U.S. addiction physicians’ low rate of naltrexone prescription. Drug Alcohol Depend.

[3] Jonas DE, Amick HR, Feltner C, Bobashev G, Thomas K, Wines R (2014). Pharmacotherapy for adults with alcohol use disorders in outpatient settings: a systematic review and meta-analysis. JAMA.

[4] Koob GF, Volkow ND (2016). Neurobiology of addiction: a neurocircuitry analysis. Lancet Psychiatry.

[5] Mason BJ, Quello S, Goodell V, Shadan F, Kyle M, Begovic A (2014). Gabapentin treatment for alcohol dependence: a randomized clinical trial. JAMA Intern Med.

[6] Kranzler HR, Van Kirk J (2001). Efficacy of naltrexone and acamprosate for alcoholism treatment: a meta-analysis. Alcohol Clin Exp Res..

[7] Pierce M, Sutterland A, Beraha EM, Morley K, van den Brink W (2018). Efficacy, tolerability, and safety of low-dose and high-dose baclofen in the treatment of alcohol dependence: a systematic review and meta-analysis. Eur Neuropsychopharmacol.

[8] Bschor T, Henssler J, Müller M, Baethge C (2018). Baclofen for alcohol use disorder-a systematic meta-analysis. Acta Psychiatr Scand.

[9] Rose AK, Jones A (2018). Baclofen: its effectiveness in reducing harmful drinking, craving, and negative mood. A meta-analysis. Addiction.

[10] de Beaurepaire R (2012). Suppression of alcohol dependence using baclofen: a 2-year observational study of 100 patients. Front Psychiatry.

[11] Addolorato G, Leggio L, Ferrulli A, Cardone S, Vonghia L, Mirijello A (2007). Effectiveness and safety of baclofen for maintenance of alcohol abstinence in alcohol-dependent patients with liver cirrhosis: randomised, double-blind controlled study. Lancet..

[12] Morley KC, Baillie A, Fraser I, Furneaus-Bate A, Dore G, Roberts M (2018). Baclofen in the treatment of alcohol dependence with or without liver disease: multisite, randomised, double-blind, placebo-controlled trial. Br J Psychiatry.

[13] Agabio R, Sinclair JM, Addolorato G, Aubin HJ, Beraha EM, Caputo F (2018). Baclofen for the treatment of alcohol use disorder: the Cagliari Statement. Lancet Psychiatry.

[14] Garbutt JC, Kampov-Polevoy AB, Gallop R, Kalka-Juhl L, Flannery BA (2010). Efficacy and safety of baclofen for alcohol dependence: a randomized, double-blind, placebo-controlled trial. Alcohol Clin Exp Res.

[15] Hauser P, Fuller B, Ho SB, Thuras P, Kern S, Dieperink E (2017). The safety and efficacy of baclofen to reduce alcohol use in veterans with chronic hepatitis C: a randomized controlled trial. Addiction.

[16] Leggio L, Zywiak WH, Edwards SM, Tidey JW, Swift RM, Kenna GA (2015). A preliminary double-blind, placebo-controlled randomized study of baclofen effects in alcoholic smokers. Psychopharmacology.

[17] First, MB, Spitzer RL, Gibbon M, Williams JBW. Structured clinical interview for DSM-IV-TR axis I disorders, research version, patient edition. (SCID-I/P) 2002; New York: Biometrics Research, New York State Psychiatric Institute.

[18] Sobell LC, Sobell MB, Leo GL, Cancilla A (1988). Reliability of a timeline followback method: Assessing normal drinkers’ reports of recent drinking and a comparative evaluation across several populations. Brit J Addictions.

[19] Flannery BA, Volpicelli JR, Pettinati HM (1999). Psychometric properties of the Penn Alcohol Craving Scale. Alcohol Clin Exp Res.

[20] Speilberger CD, Gorsuch RL, Lushene RE. The state trait anxiety inventory manual. 1969; Palo Alto: Consulting Psychologists Press.

[21] Sheehan D, Janavs J, Baker R, Harnett-Sheehan K, Knapp E, Sheenan, M. Mini International Neuropsychiatric Interview (M.I.N.I.). 1999; Tampa: University of South Florida.

[22] Pettinati HM, Weiss RD, Dundon W, Miller WR, Donovan D, Ernst DB (2005). A structured approach to medical management: a psychosocial intervention to support pharmacotherapy in the treatment of alcohol dependence. J Stud Alcohol Suppl.

[23] Falk D, Wang XQ, Liu L, Fertig J, Mattson M, Ryan M (2010). Percentage of subjects with no heavy drinking days: evaluation as an efficacy endpoint for alcohol clinical trials. Alcohol Clin Exp Res.

[24] Gibbons RD, Hedeker D, Elkin I, Waternaux C, Kraemer HC, Greenhouse JB (1993). Some conceptual and statistical issues in analysis of longitudinal psychiatric data. Arch Gen Psychiatry.

[25] Garbutt JC, Kampov-Polevoy AB, Kalka-Juhl L, Gallop RJ (2016). Sweet liking phenotype and craving for alcohol moderate response to naltrexone treatment in alcohol dependence: a randomized, placebo-controlled trial. JAMA Psychiatry.

[26] Hedeker D, Gibbons RD (1997). Application of random-effects pattern-mixture models for missing data in longitudinal studies. Psychological Methods.

[27] Gastfriend DR, Garbutt JC, Pettinati HM, Forman RF (2007). Reduction in heavy drinking as a treatment outcome in alcohol dependence. J Subst Abus Treat.

[28] Addolorato G1, Caputo F, Capristo E, Domenicali M, Bernardi M, Janiri L,A (2002). Baclofen efficacy in reducing alcohol craving and intake: a preliminary double-blind randomized controlled study. Alcohol Alcohol.

[29] Anton RF, O’Malley SS, Ciraulo DA, Cisler RA, Couper D, Donovan DM (2006). Combined pharmacotherapies and behavioral interventions for alcohol dependence: the COMBINE study: a randomized controlled trial. JAMA.

[30] Mason BJ, Goodman AM, Chabac S, Lehert P (2006). Effect of oral acamprosate on abstinence in patients with alcohol dependence in a double-blind, placebo-controlled trial: the role of patient motivation. J Psychiatr Res.

[31] Rigal L, Legay Hoang L, Alexandre-Dubroeucq C, Pinot J, Le Jeunne C, Jaury P (2015). Tolerability of high-dose baclofen in the treatment of patients with alcohol disorders: a retrospective study. Alcohol Alcohol.

[32] Leggio L, Zywiak WH, Edwards SM, Tidey JW, Swift RM, Kenna GA (2015). A preliminary double-blind, placebo-controlled randomized study of baclofen effects in alcoholic smokers. Psychopharmacology.

[33] Lyon JE, Khan RA, Gessert CE, Larson PM, Renier CM (2011). Treating alcohol withdrawal with oral baclofen: a randomized, double-blind, placebo-controlled trial. J Hosp Med.

[34] Addolorato G, Leggio L, Abenavoli L, Agabio R, Fabio Caputo F, Capristo E (2006). Baclofen in the treatment of alcohol withdrawal syndrome: a comparative study vs diazepam. Am J Med.

[35] Saitz R (2007). Baclofen for alcohol withdrawal: not comparable to the gold standard (benzodiazepines). Am J Med.

[36] Stephens J, Moore C, Stepanek KV, Garbutt JC, Starke B, Liles A (2018). Implementation of a process for initiating naltrexone in patients hospitalized for alcohol detoxification or withdrawal. J Hosp Med.

[37] Vengeliene V, Takahashi TT, Dravolina OA, Belozertseva I, Zvartau E, Bespalov AY (2018). Efficacy and side effects of baclofen and the novel GABAB receptor positive allosteric modulator CMPPE in animal models for alcohol and cocaine addiction. Psychopharmacology.

[38] Loi B1, Maccioni P, Lobina C, Carai MAM, Gessa GL, Thomas AW (2013). Reduction of alcohol intake by the positive allosteric modulator of the GABA(B) receptor, rac-BHFF, in alcohol-preferring rats. Alcohol..

[39] Maccioni P, Colombo G (2019). Potential of GABA B receptor positive allosteric modulators in the treatment of alcohol use disorder. CNS Drugs.

[40] Beraha EM, Salemink E, Goudriaan AE, Bakker A, de Jong D, Smits N (2016). Efficacy and safety of high-dose baclofen for the treatment of alcohol dependence: a multicentre, randomised, double-blind controlled trial. Eur Neuropsychopharmacol.

