# Supplementary material for: Efficacy and tolerability of baclofen in a U.S. community population with alcohol use disorder: a dose-response, randomized, controlled trial
Source: Neuropsychopharmacology. 2021 Jun 21;46(13):2250–6. doi: 10.1038/s41386-021-01055-w (PMC8580979; doi:10.1038/s41386-021-01055-w)
Supplement: Supplementary file 1 — Protocol [file 41386_2021_1055_MOESM1_ESM.doc]

**Clinical Research Protocol**

**Efficacy and Safety of High-Dose Baclofen for Alcohol Dependence**

| **Regulatory Sponsor:** | James C. Garbutt, MD  10101 Neurosciences Hosp  Campus Box 7160  Chapel Hill, NC  27599-7160  Phone:(919) 966-4652 |
| --- | --- |
| **Funding Sponsor:** | The National Institutes of Health (NIH) |
| **Study Product:** | Baclofen / placebo |
| **IRB Number:** | 12-1743 |
|  |  |

**Table of Contents**

1 Introduction [1](#__RefHeading___Toc366665946)

1.1 Background [1](#__RefHeading___Toc366665947)

1.2 Investigational Agent [2](#__RefHeading___Toc366665948)

1.3 Preclinical Data [2](#__RefHeading___Toc366665949)

1.4 Clinical Data to Date [3](#__RefHeading___Toc366665950)

1.5 Dose Rationale and Risk/Benefits [6](#__RefHeading___Toc366665951)

2 Study Objectives [6](#__RefHeading___Toc366665952)

3 Study Design [7](#__RefHeading___Toc366665953)

3.1 General Design [7](#__RefHeading___Toc366665954)

4 Subject Selection and Withdrawal [7](#__RefHeading___Toc366665955)

4.1 Inclusion Criteria [7](#__RefHeading___Toc366665956)

4.2 Exclusion Criteria [7](#__RefHeading___Toc366665957)

4.3 Subject Recruitment and Screening [8](#__RefHeading___Toc366665958)

4.4 Early Withdrawal of Subjects [8](#__RefHeading___Toc366665959)

4.4.1 Data Collection and Follow-up for Withdrawn Subjects [9](#__RefHeading___Toc366665960)

5 Study Drug [9](#__RefHeading___Toc366665961)

5.1 Description [9](#__RefHeading___Toc366665962)

5.2 Treatment Regimen [9](#__RefHeading___Toc366665963)

5.3 Method for Assigning Subjects to Treatment Groups [10](#__RefHeading___Toc366665964)

5.4 Preparation and Administration of Study Drug [10](#__RefHeading___Toc366665965)

5.5 Packaging [10](#__RefHeading___Toc366665966)

5.6 Blinding of Study Drug (if applicable) [11](#__RefHeading___Toc366665967)

5.6.1 Receipt of Drug Supplies [11](#__RefHeading___Toc366665968)

5.6.2 Dispensing of Study Drug [11](#__RefHeading___Toc366665969)

5.6.3 Return or Destruction of Study Drug [11](#__RefHeading___Toc366665970)

6 Study Procedures [11](#__RefHeading___Toc366665971)

7 Statistical Plan [17](#__RefHeading___Toc366665972)

7.1 Sample Size Determination [17](#__RefHeading___Toc366665973)

7.2 Statistical Methods [18](#__RefHeading___Toc366665974)

8 Safety and Adverse Events [19](#__RefHeading___Toc366665975)

8.1 Definitions [19](#__RefHeading___Toc366665976)

8.2 Recording of Adverse Events [21](#__RefHeading___Toc366665977)

8.3 Reporting of Serious Adverse Events and Unanticipated Problems [21](#__RefHeading___Toc366665978)

8.3.1 Investigator reporting: [22](#__RefHeading___Toc366665979)

8.3.2 Sponsor reporting: Notifying the FDA [22](#__RefHeading___Toc366665980)

8.4 Unblinding Procedures [23](#__RefHeading___Toc366665981)

8.5 Stopping Rules [23](#__RefHeading___Toc366665982)

8.6 Medical Monitoring [24](#__RefHeading___Toc366665983)

8.6.1 Data and Safety Monitoring Board [24](#__RefHeading___Toc366665984)

9 Data Handling and Record Keeping [24](#__RefHeading___Toc366665985)

9.1 Confidentiality [24](#__RefHeading___Toc366665986)

9.2 Source Documents [25](#__RefHeading___Toc366665987)

9.3 Data Collection [25](#__RefHeading___Toc366665988)

9.4 Records Retention [25](#__RefHeading___Toc366665989)

10 Study Monitoring, Auditing, and Inspecting [25](#__RefHeading___Toc366665990)

10.1 Study Monitoring Plan [25](#__RefHeading___Toc366665991)

10.2 Auditing and Inspecting [26](#__RefHeading___Toc366665992)

11 Ethical Considerations [26](#__RefHeading___Toc366665993)

12 Study Finances [27](#__RefHeading___Toc366665994)

12.1 Funding Source [27](#__RefHeading___Toc366665995)

12.2 Conflict of Interest [27](#__RefHeading___Toc366665996)

12.3 Subject Stipends or Payments [27](#__RefHeading___Toc366665997)

13 Publication Plan [27](#__RefHeading___Toc366665998)

14 References [27](#__RefHeading___Toc366665999)

**ABSTRACT**

Alcohol dependence (AD) is a common problem with significant health consequences. Treatment of AD is evolving to include both counseling methods and medications. Several medications have been discovered, that show efficacy in AD, e.g. naltrexone, acamprosate. However, the overall effect of existing medications is modest leaving a clear need for the development of new pharmacotherapies. The GABAB receptor agonist baclofen has attracted attention as a potential new medication for AD based on preclinical data and early clinical trials. Baclofen is an FDA approved medication with an excellent safety profile even for patients with liver cirrhosis—a not uncommon consequence of AD. Questions have arisen with regards to the efficacy of baclofen and whether higher doses of baclofen are safe and more effective than the prior tested dose of 30 mg/ day. There is emerging evidence that severity of dependence is positively associated with baclofen response. The main goal of the present proposal is to test the efficacy and safety of 30 mg/d and 90 mg/d of baclofen compared to placebo controlling for severity of dependence as assessed by drinks/drinking day. A primary secondary goal will examine for an anxiolytic effect of baclofen.

This study proposes to enroll 120 men and women with AD in a randomized, placebo-controlled trial to include at least 60 individuals with more severe AD (≥14 drinks/drinking day for men; ≥10 drinks/drinking day for women) with randomization to baclofen or placebo balanced for this variable. Baclofen will be titrated to 10 mg t.i.d over 3 days and to 30 mg t.i.d over 12 days and maintained at that level for 12 weeks and then downtitrated for a total study time of 16 weeks. Medical Management will be provided to encourage progress towards drinking goals and to enhance retention and compliance. Drinking patterns, anxiety levels, sleep patterns, craving for alcohol, gamma-glutamyl transferase (GGT) and carbohydrate deficient transferring (CDT) will be assessed. Trough blood levels of R & S-baclofen will be assessed in all individuals at week 4.

In summary, the present proposal is innovative and of clinical significance as it will test and compare standard and high-dose baclofen for efficacy and safety in individuals with AD. The proposal is adequately powered to test the primary hypothesis and provides good power to assess whether drinks/drinking day is predictive of baclofen response. Adequate power is also present to examine the anxiolytic effect of baclofen. Ascertaining the effects of standard and high-dose baclofen, the predictive value of heavy drinking on baclofen response and the anxiolytic effect of baclofen are important goals towards determining whether baclofen has true value for the clinical management of the patient with alcohol dependence.

# Introduction

This document is a protocol for a human research study. This study is to be conducted according to US and international standards of Good Clinical Practice (FDA Title 21 part 312 and International Conference on Harmonization guidelines), applicable government regulations and Institutional research policies and procedures.

## Background

Alcohol misuse including alcohol dependence (AD) is a major public health problem in the United

States. The Center for Disease Control places alcohol as the number three cause of preventable deaths following nicotine use and overweight (Mokdad et al, 2004). The economic costs of alcohol misuse are estimated at about $225 billion per year (Bouchery et al, 2011). Epidemiological studies show that AD effects on the order of 5-6% of men and 2-3% of women in the United States in a given 12 month period (Grant et al, 2004)—in sum, AD is a common disorder with major health and economic consequences

Despite the prevalence and destructiveness of AD, many patients with AD do not receive treatment and, often, treatment is not as effective as either the patient or the clinician desire. Considerable effort has been mounted on the part of government agencies, professional societies, health care institutions, clinicians and lay groups and individuals to expand and improve treatment for AD. These efforts have taken many forms including educating the public and clinicians through campaigns, encouraging screening for alcohol problems in primary care settings, and investigating new psychosocial and pharmacologic treatments.

Treatment for AD can be very effective-- Miller et al (2001) note that on the order of 25% of individuals maintain sobriety at one year after several of a variety of treatment interventions. However, many patients do not do well in treatment and only 25% or so of those with AD ever get treatment. These latter statistics have encouraged the development of improved treatment approaches including efforts to identify treatments targeted towards possible subgroups of AD, e.g AD with anxiety symptoms. These efforts have been quite successful as two new medications, naltrexone and acamprosate, have been approved for AD by the FDA in the past 20 years. Nevertheless, naltrexone and acamprosate have small effect sizes (Kranzler and Van Kirk, 2001) when considered across the broad population of all patients with AD and their utilization has been disappointingly limited.

To move the treatment of AD forward there is a compelling need to find new medications that are both effective and well tolerated. Furthermore, it would be very helpful for clinicians to have access to medications that target not only drinking behavior but also other aspects of the AD syndrome such as anxiety that are commonly found in patients with AD and contribute to relapse (Kushner et al, 2000). Achieving the latter goal would represent a step towards personalized medicine in AD.

A body of preclinical and clinical evidence has arisen over the past 30 years that indicates that the

GABAB agonist baclofen may have therapeutic value in AD. Baclofen has advantages as a possible therapeutic innovation for AD as it is approved by the FDA and has an extensive track record in human use. Its safety profile is quite good--even with intentional overdoses most patients fully recover (see Wall et al, 2006). The drug is rapidly absorbed after oral administration and is widely distributed throughout the body. Biotransformation is low and the drug is predominantly excreted in the unchanged form by the kidneys, which makes it safe to use in AD patients with compromised liver function (Addolorato et al., 2007). Another beneficial property of baclofen is that tolerance does not seem to occur to any significant degree — baclofen retains its therapeutic effects even after many years of continued use (Gaillard 1977).

Therefore, if baclofen does indeed demonstrate efficacy in AD, it would provide clinicians with another readily available tool to treat the AD patient. Furthermore, there is solid preclinical and clinical evidence that baclofen also can target anxiety symptoms in AD and may even reduce the likelihood of their development (see Knapp et al, 2007). If true, baclofen could be of considerable value as a means to help manage anxiety symptoms in the AD patient—symptoms which can be difficult to manage and which can trigger relapse. Baclofen is particularly interesting in this regard as, unlike other anxiolytics such as the benzodiazepines, it does not have clear addictive properties and appears relatively safe when combined with alcohol. The purpose of the present proposal is to investigate the efficacy and tolerability of baclofen for alcohol dependence with attention to several key issues including baclofen dose, severity of dependence and severity of coexisting anxiety symptoms.

## Investigational Agent

Lioresal, baclofen USP, is a muscle relaxant and antispastic, available as 10-mg and 20-mg tablets for oral administration. Its chemical name is 4-amino-3-(4-chlorophenyl)- butanoic acid, and its structural formula is Baclofen USP is a white to off-white, odorless or practically odorless crystalline powder, with a molecular weight of 213.66. It is slightly soluble in water, very slightly soluble in methanol, and insoluble in chloroform. Inactive Ingredients. Cellulose compounds, magnesium stearate, povidone, and starch.

The precise mechanism of action of Lioresal is not fully known. Lioresal is capable of inhibiting both monosynaptic and polysynaptic reflexes at the spinal level, possibly by hyperpolarization of afferent terminals, although actions at supraspinal sites may also occur and contribute to its clinical effect. Although Lioresal is an analog of the putative inhibitory neurotransmitter gamma-aminobutyric acid (GABA), there is no conclusive evidence that actions on GABA systems are involved in the production of its clinical effects. In studies with animals, Lioresal has been shown to have general CNS depressant properties as indicated by the production of sedation with tolerance, somnolence, ataxia, and respiratory and cardiovascular depression. Lioresal is rapidly and extensively absorbed and eliminated. Absorption may be dose-dependent, being reduced with increasing doses. Lioresal is excreted primarily by the kidney in unchanged form and there is relatively large intersubject variation in absorption and/or elimination.

## Preclinical Data

Effect of Baclofen on Ethanol Intake and Ethanol-Induced Dopamine Activation

Over 30 years ago Cott et al (1976) reported that baclofen eliminated ethanol-induced locomotor stimulation and proposed that this effect occurred via inhibition of dopamine release. As they noted then“These findings also indicate a potential interaction between GABA-like drugs and alcohol in man, and may be Research Strategy Page 36 Principal Investigator/Program Director (Last, first, middle): Garbutt, James, C of heuristic value in the treatment of chronic alcoholism.” Since then, studies completed over the past 20 years have shown that baclofen reduces alcohol intake in several animal models and modifies behavioral actions of ethanol. As reviewed by Maccioni and Colombo (2009), baclofen: 1) reduces acquisition and maintenance of alcohol drinking; 2) counteracts the alcohol deprivation effect; 3) suppresses the reinforcing effect of alcohol in an operant model; 4) suppresses alcohol-induced locomotor activity. The underlying neuropharmacology of these actions is still being elucidated but one consistent finding is that baclofen and other GABAB agonists act in the ventral tegmental area to counteract ethanol-induced dopamine activation which would be expected to counteract ethanol reinforcement and locomotor activation (see Maccioni et al, 2009). However, it is of interest that baclofen, unlike classic GABAA agonists, counteracts ethanol-induced locomotor stimulation without enhancing motor-impairment (Holstein et al, 2009). This latter observation led Holstein et al (2009) to conclude “GABAB receptor agonists, at carefully titrated doses that minimize side effects, may hold greater promise as treatments for alcohol use disorders [than GABAA agonists] because of their reduced negative interaction effects with ethanol”. Therefore, based on these preclinical findings the hypothesis could be put forward that baclofen may counteract ethanol consumption and/or the urge to consume ethanol, in part, secondary to an inhibitory effect on classical dopamine motivational systems.

Effect of Baclofen on Anxiety

Baclofen and other GABAB agonists have been found to reduce anxiety in a number of animal behavioral paradigms including separation-induced anxiety, punished responding, and the elevated T-maze leading to the conclusion that activation of GABAB receptors has potential for the clinical management of anxiety (see Cryan and Kaupmann, 2005). There has been limited clinical investigation of this hypothesis though Breslow et al (1989) in a placebo-controlled trial in patients with panic disorder found that 30 mg of baclofen was superior to placebo in reducing both panic attacks and anxiety. Additionally, Drake et al (2003) reported that 80 mg of open-label baclofen was effective in reducing hyperarousal, avoidance and anxiety in patients with post-traumatic stress disorder, an anxiety disorder. Overall, evidence indicates that baclofen and GABAB agonists have anxiolytic properties that may have value for treatment

In summary, the preclinical evidence indicates that baclofen can reduce alcohol consumption in a variety of behavioral models. Of interest is that it appears to have a two-fold action: 1) counteracting the classic alcohol-induced reinforcement mediated through limbic-cortical DA systems and 2) counteracting alcohol-induced anxiogenic actions through a not fully understood neuropharmacological mechanism. This two-fold action is of considerable interest to the clinic as it suggests that baclofen may have value in reducing both alcohol use and anxiety symptoms.

## Clinical Data to Date

To date, seven clinical trials of baclofen in AD targeting drinking behavior have been completed and published including four placebo-controlled, double-blind trials. These results are summarized in Table 1

These trials show that the overall number of individuals with AD treated with baclofen in all published clinical trials is quite modest, n=181, with an additional n=138 receiving placebo. Nevertheless, the data are provocative as two placebo-controlled trials (Addolorato et al, 2002; 2007) show a robust effect of baclofen for enhancing abstinence and reducing drinking quantities in AD and a third trial (Addolorato et al, 2011) shows a baclofen effect on reducing drinks/day. However, the trial by Garbutt et al (2010) did not detect an effect for baclofen on abstinence or heavy drinking though a significant effect on anxiety was found.

In an effort to understand the discrepancies across these trials, Leggio et al (2010) completed a comparison of the Garbutt et al (2010) trial to the Addolorato et al (2002) trial (non-cirrhotics) and found some substantial differences between the trials as outlined here:.

-The Addolorato et al (2002) trial enrolled more severe alcoholic patients with:

1) twice the number of Drinks per Drinking Day (DDD) (14.2 vs 7.0 respectively)

2) much higher rates of alcohol withdrawal symptoms Clinical Institute Withdrawal Assessment for Alcohol Revised (CIWA-Ar, Sullivan et al., 1989) scores of 12.3 vs. 2.4)

When our own data (Garbutt et al., 2010) was reanalyzed, it was shown that the effect of baclofen on abstinent days was stronger in patients with more severe AD (as measured by DDD levels at admission; see Preliminary Data in Approach, C.2)

The Addolorato et al (2002) patients were uniformly committed to abstinence whereas only 24% of those in the Garbutt et al (2010) trial had abstinence as a goal. There is evidence that motivation for abstinence can positively impact response to medication in AD, see Mason et al. (2006.)

In the Addolorato et al (2002) trial it is also noted that baclofen and placebo were entrusted to “a referred family member”, which likely enhances compliance (compliance rate was universally >90%; Leggio, personal communication). As has been shown in early work by Volpicelli et al (1997), naltrexone compliance is a significant factor affecting outcomes. In Garbutt et al., (2010) study compliance was in the 48-96%range.

In summary, the comparison across the early clinical trials of baclofen suggests that severity of alcohol dependence may be a key factor in predicting response. Accordingly, for our proposed trial we will recruit a population with heavier drinking to more closely match the population studied by Addolorato et al (2002) and we will randomize within treatment group on Drinks/Drinking Day using a cutoff of ≥ 14 for men and ≥10 for women;.

**High-Dose Baclofen for Alcohol Dependence**:

Several case reports have been published on the use of high dose baclofen for alcohol dependence. The first report was that of Amiesen (2005), a physician with AD and an anxiety disorder who titrated himself to a dose of baclofen of 270 mg/d and then reduced the dose to 120 mg/d. He achieved and maintained sobriety and reported marked improvement in anxiety, reduced muscle tension, improved sleep and a complete suppression of craving for alcohol. Prior treatments with naltrexone, acamprosate, disulfiram and topiramate had not been effective. The second case report (Bucknam, 2007) described a patient with alcohol dependence who had failed treatments with naltrexone, acamprosate and topiramate. Treatment with baclofen up to 140 mg/d led to marked improvement in alcohol use, a blunting of the euphorigenic effect of alcohol and a sense of mild relaxation. Pastor et al (2012) recently reported on four cases of treatment resistant AD that responded to baclofen doses of 75-125 mg/d. Case reports do not provide evidence of efficacy but can provide clues that should be pursued in a more systematic fashion.

Recently, Addolorato et al (2011) published the first placebo-controlled pilot study, n=42, in alcohol dependence comparing 30 mg of baclofen to a higher dose of 60 mg/d. They did not find a baclofen effect on heavy drinking or abstinent days but, in a secondary analysis, found that baclofen led to greater reductions in drinks/day than did placebo (p<.0001) and that 60 mg of baclofen had a greater effect than 30 mg/d (p=0.02). The overall effect was modest but the dose response effect is supportive of the idea that higher doses of baclofen should be investigated

**Anxiety Symptoms/Disorders and Alcohol Use Disorders: Relevance to Baclofen**

A strong relationship between anxiety symptoms and anxiety disorders and alcohol use disorders has been established in the literature for decades. The relationship is quite complex and has been the subject of debate. As noted by Kushner et al (2000) in their review, “Findings converge on the conclusion that anxiety disorder and alcohol disorder can both serve to initiate the other….” Twelve month prevalence rates of DSMIV diagnosed anxiety disorders have been reported to be 23.5% in individuals with AD compared to 10.4% in individuals without a substance-use disorder (Grant et al, 2004) indicating the high rate of anxiety disorders in

AD. In clinical and laboratory studies, alcohol is shown to have both an acute anxiolytic effect and a delayed anxiogenic effect (see Kushner et al, 2000). This led Kushner et al (2000) to propose a feed-forward cycle where the initial anxiolytic effect of alcohol is followed by an anxiogenic effect which then promotes the use of alcohol for its anxiolytic effect.

The relationship between anxiety disorders, anxiety symptoms and alcohol use disorders continues to be studied but it seems clear that preexisting anxiety is a risk-factor for development of AD, that anxiety symptoms can contribute to relapse and that alcohol has both acute anxiolytic and delayed anxiogenic effects.

Therefore, methods to reduce the development of anxiety symptoms and to improve the management of anxiety symptoms in patients with AD are important clinical issues. Baclofen’s overall anxiolytic effects (Cryan and Kaupmann, 2005), its ability to counteract ethanol-induced anxiety (Knapp et al, 2007) and its evidence of antianxiety efficacy in early clinical trials in AD (Addolorato et al, 2002; Garbutt et al, 2010) all point to the conclusion that baclofen may be particularly valuable in the management of the AD patient with anxiety symptoms. However, to carefully test this hypothesis it is necessary to conduct a placebo-controlled trial in individuals with AD.

## Dose Rationale and Risk/Benefits

We have chosen to utilize a 90 mg dose of oral baclofen per day for the high-dose arm of the trial because this dose is in the range of the higher baclofen doses shown to be safe in a trial of nicotine dependence (Franklin et al, 2009) and in an interaction study with ethanol (Evans and Bisaga, 2009). The Franklin et al (2009) trial used an 80 mg/d dose given as 20 mg four times per day. We propose a 30 mg dose given three times per day (morning, late afternoon, bedtime) which should be logistically easier for participants then a four times a day schedule.

Loss of control over alcohol consumption and the development of tolerance to alcohol are key components of the AD syndrome. One indicator of loss of control and tolerance is the amount of alcohol an individual consumes in a day. All clinical trials in AD, including the present trial, require that some minimal threshold of alcohol consumption is ongoing in participants in order to meet inclusion criteria for the trial. Commonly, some minimum number of heavy drinking days (defined as ≥5 standard drinks/d for a man and ≥4 standard drinks/d for a woman) per week or per month is required. Whereas this requirement sets a lower bound for alcohol consumption, the range above the lower bound is large. Therefore, though participants in the prior placebo-controlled clinical trials of baclofen all met criteria for AD as defined by DSM-III-R or DSM-IV, the average number of DDD differed by a factor of two--14 drinks/drinking day for Addolorato et al (2002) vs 7 drinks/drinking day for Garbutt et al (2010). Therefore, we will require 50% of our study population to have a minimum of an average of ≥14 DDD for men and ≥10 DDD for women in the 30 days prior to screening and will control for this factor by including it as a randomization variable for baclofen/placebo

# Study Objectives

Primary :

- To test the efficacy and safety of 30 mg/d and 90 mg/d of baclofen compared to placebo for individuals with alcohol dependence.

Secondary:

- To test the hypothesis that a history of heavy drinking as assessed by average drinks/drinking day in the 30 days preceding screening predicts baclofen response in alcohol dependence.
- To test the hypothesis that baclofen, 30 mg/d and 90 mg/d, is superior to placebo in reducing anxiety in alcohol dependence.

# Study Design

## General Design

This is a Phase II, randomized, double blind, placebo controlled trial of baclofen for the treatment of alcohol dependence. The study proposes to enroll 120 men and women with AD in a randomized, placebo-controlled trial to include at least 60 individuals with high levels of drinking (≥14 drinks/drinking day for men; ≥10 drinks/drinking day for women) with randomization to baclofen or placebo balanced for this variable. Baclofen will be titrated to 10 mg t.i.d over 3 days and to 30 mg t.i.d over 12 days and maintained at that level for 12 weeks and then down-titrated for a total study time of 16 weeks. Medical Management will be provided to encourage progress towards drinking goals and to enhance retention and compliance. Drinking patterns, anxiety levels, sleep patterns, craving for alcohol, gamma-glutamyl transferase (GGT) and carbohydrate deficient transferrin (CDT) will be assessed.

# Subject Selection and Withdrawal

## Inclusion Criteria

1. Men and women between the ages of 18 and 65 meeting DSM-IV criteria for current alcohol dependence.
2. More than 14 drinks (women) or 21 drinks (men) per week including at least 2 heavy drinking days (men > 5 drinks/day; women > 4 drinks/day) per week in the 30-day period prior to screening. In addition we will recruit 50% of individuals who have a mean of ≥14 drinks/drinking day (men) or ≥10 drinks/drinking day (women) in the 30 days prior to screening.
3. Ability to understand and sign written informed consent.
4. Must have a 0.0 gms/dl breathalyzer reading on the day of screening and 0.0 gms/dl on the day of randomization.
5. Express a desire to achieve abstinence or to greatly reduce alcohol consumption
6. Must have a stable residence and be able to identify an individual who could contact participant if needed.

## Exclusion Criteria

1. Clinically significant medical disease that might interfere with the evaluation of the study medication or present a safety concern (e.g., renal insufficiency, cirrhosis, unstable hypertension, diabetes mellitus, seizure disorder). Clinically significant psychiatric illness including any psychotic disorder, bipolar disorder, severe depression, or suicidal ideation.
2. Other substance abuse or dependence disorder other than nicotine or alcohol or cannabis abuse. Occasional use of cocaine is acceptable.
3. Concurrent use of any psychotropic medication including antidepressants, mood stabilizers, antipsychotics, anxiolytics, stimulants, or hypnotics with the exception of stable doses of antidepressants for one month. Concurrent use of anticonvulsants, insulin, or oral hypoglycemics.
4. Prior history of adverse reaction to baclofen.
5. Estimated Glomerular Filtration Rate < age norm.
6. AST, or ALT > 5 times ULN or bilirubin > 1.5 X ULN.
7. Positive urine toxicology screen with the exception of cannabis. Individuals with positive cannabis screens will be excluded only if they have a history of cannabis dependence.
8. Pregnant women and women of childbearing potential who do not practice a medically acceptable form of birth control (oral or depot contraceptive, or barrier methods such as diaphragm or condom with spermicidal).
9. Women who are breastfeeding.
10. Individuals requiring inpatient treatment or more intense outpatient treatment for their alcohol dependence.
11. Participation in any clinical trial within the past 60 days.
12. Court-mandated participation in alcohol treatment or pending incarceration.

## Subject Recruitment and Screening

It is anticipated that 120 participants can be recruited within 48 months based on prior rates of enrollment for alcohol studies conducted by the proposed study team and the awareness that the current proposal will study individuals over a longer period of time and needs to recruit a minimum number of individuals with heavy drinking and with high anxiety. In our recently completed placebo study of baclofen we were able to recruit 80 subjects over 19 months with 75% trial retention. Retention is an important objective of the trial with our target being a minimum of 75% of subjects completing the trial. Medical Management and the regular meetings with our research coordinator during study visits have proven useful in encouraging retention and compliance with medication and we have averaged 75-85% retention for most trials.

Potential participants will be recruited from the Raleigh, Durham, and Chapel Hill areas by screening patients at Freedom House and via advertising and referrals. Freedom House is a local publically funded detoxification center. Approximately 350 alcohol dependent individuals per year are admitted to Freedom House. These individuals have been screened to minimize risk of more serious withdrawal problems such as DTs or seizures and individuals with serious psychiatric problems such as suicidal behavior or psychosis or serious medical problems that require acute care are not admitted. Patients at Freedom House receive medical or social detoxification depending on symptom severity. A CIWA-Ar driven protocol using lorazepam has been utilized there. The average length of stay at Freedom House is 4 days. This population represents treatment seeking individuals who are more likely to have higher levels of physical dependence than our traditional trial population recruited by advertising. This is a very important point as this population may be the one that responds best to baclofen (see Leggio et al, 2010).

Individuals at Freedom House will be screened in person whereas those who respond to advertisements will have a preliminary telephone screening conducted by the study coordinator. Individuals who appear eligible, as determined by the investigative team, will be scheduled to come to the UNC Hospitals for more comprehensive screening. Prior to full screening, individuals will read and sign the informed consent and be given a copy for their records.

## Early Withdrawal of Subjects

Subjects can withdraw from this study at any time, without penalty. The investigators also have the right to stop subject’s participation at any time.

A subject who experiences a serious adverse event thought to be possibly or probably related to study medication will have their medication tapered and stopped. It is preferable to taper baclofen then stopping it abruptly. If a subject is having moderate side-effects the medication can be reduced and side-effects monitored. If a subject continues to have side-effects the medication may be tapered and stopped. We will make every effort to maintain subjects in the trial as long as safety is adhered to. If a subject does not comply with medication instructions we may need to discontinue medication for safety purposes. We will follow subjects off medication to collect outcome data such as drinking behavior.

### Data Collection and Follow-up for Withdrawn Subjects

Any data collected up to the time of withdrawal will be kept, as well as any follow-up information related to subject safety.

# Study Drug

## Description

Lioresal, baclofen USP, is a muscle relaxant and antispastic, available as 10-mg and 20-mg tablets for oral administration. Baclofen USP is a white to off-white, odorless or practically odorless crystalline powder, with a molecular weight of 213.66. It is slightly soluble in water, very slightly soluble in methanol, and insoluble in chloroform.

Inactive Ingredients. Cellulose compounds, magnesium stearate, povidone, and starch.

The precise mechanism of action of Lioresal is not fully known. Lioresal is capable of inhibiting both monosynaptic and polysynaptic reflexes at the spinal level, possibly by hyperpolarization of afferent terminals, although actions at supraspinal sites may also occur and contribute to its clinical effect. Although Lioresal is an analog of the putative inhibitory neurotransmitter gamma-aminobutyric acid (GABA), there is no conclusive evidence that actions on GABA systems are involved in the production of its clinical effects. In studies with animals, Lioresal has been shown to have general CNS depressant properties as indicated by the production of sedation with tolerance, somnolence, ataxia, and respiratory and cardiovascular depression. Lioresal is rapidly and extensively absorbed and eliminated. Absorption may be dose-dependent, being reduced with increasing doses. Lioresal is excreted primarily by the kidney in unchanged form and there is relatively large inter subject variation in absorption and/or elimination.

## Treatment Regimen

It is important to uptitrate and downtitrate the dose of baclofen to minimize side-effects. We will parallel the titration schedule used by Franklin et al (2009) in their study of 20 mg of baclofen given four times per day. In the latter study, baclofen was well tolerated without significant differences between baclofen and placebo groups--for the chief side-effect of sedation, the baclofen group had 20 mild, 4 moderate and 0 severe reports of sedation compared to 4 reports of mild sedation and 0 reports of either moderate or severe sedation with placebo (Franklin et al, 2009). Our titration schedule for the 90 mg/d dose is shown in Table 1. The 30 mg/d arm will reach 30 mg/d at day 3 and titrate down starting at day 101.

## Method for Assigning Subjects to Treatment Groups

Randomizaton blocks will be prepared by the statistician and provided to the Investigational Drug Services. A minimization random assignment procedure will be used to match subjects on the key moderator (DDD) (< 14 DDD or ≥14 DDD for men and <10 DDD or ≥10 DDD for women. Randomization will be linked to sequential study number which subjects receive as they enroll in the study.

## Preparation and Administration of Study Drug

UNC Hospital’s Investigational Drug Services (IDS) will purchase baclofen tablets in 10 mg and 20 mg strengths and create opaque baclofen capsules and matching placebo that will be inserted into blister packs. The 10 mg baclofen/placebo capsule and the 20 mg baclofen/placebo capsule will be color coded so physicians can make finer dose adjustments if needed. All used and unused blister cards will be returned to the IDS. Dr. Robert Gallop, the study biostatistician, will prepare the randomization schedule and include blocking by high/low DDD (≥14 for men and (≥10 DDD for women). The randomization schedule will be given to IDS personnel; investigators will not have access to this information. If a medical or other emergency necessitates breaking the blind, IDS personnel will provide the identity of the medication to the PI or another medical study team member.

## Packaging

Tablets 10 mg – oval, white, scored (imprinted Lioresal on one side and 10 twice on the scored side)

Bottles of 100………………………………………………….NDC 0028-0023-01

Unit Dose (blister pack)

Box of 100 (strips of 10)………………………………………NDC 0028-0023-61

Tablets 20 mg – capsule - shaped, white, scored (imprinted Lioresal on one side and 20 twice on the scored side)

Bottles of 100………………………………………………….NDC 0028-0033-01

Unit Dose (blister pack)

Box of 100 (strips of 10)………………………………………NDC 0028-0033-61

Do not store above 30ºC (86ºF). Dispense in tight container (USP).

## Blinding of Study Drug (if applicable)

The study biostatistician, will prepare the randomization schedule and include blocking by high/low DDD (≥14 for men and (≥10 DDD for women). The randomization schedule will be given to IDS personnel; investigators will not have access to this information. If a medical or other emergency necessitates breaking the blind, IDS personnel will provide the identity of the medication to the PI or another medical study team member.

### Receipt of Drug Supplies

Upon receipt of the of the study treatment supplies, an inventory must be performed and a drug receipt log filled out and signed by the person accepting the shipment. It is important that the designated study staff counts and verifies that the shipment contains all the items noted in the shipment inventory. Any damaged or unusable study drug in a given shipment (active drug or comparator) will be documented in the study files.

### Dispensing of Study Drug

The Investigational Drug Service (IDS) at UNC will be responsible for dispensing baclofen or placebo for this trial.

Participants will record their pill taking in calendar-style diaries that will be provided and collected at each visit. Additionally, baclofen and placebo will be distributed in blister packs that will be returned to the study coordinator, who will reconcile any unused medication from the returned blister packs with participants’ diary records.

### Return or Destruction of Study Drug

At the completion of the study, there will be a final reconciliation of drug shipped, drug consumed, and drug remaining. This reconciliation will be logged on the drug reconciliation form, signed and dated. Any discrepancies noted will be investigated, resolved, and documented prior to return or destruction of unused study drug. Drug destroyed on site will be documented in the study files.

# Study Procedures

Potential participants will be recruited from the Raleigh, Durham, and Chapel Hill areas by screening patients at Freedom House and via advertising and referrals. Freedom House is a local publically funded detoxification center. Approximately 350 alcohol dependent individuals per year are admitted to Freedom House. These individuals have been screened to minimize risk of more serious withdrawal problems such as DTs or seizures and individuals with serious psychiatric problems such as suicidal behavior or psychosis or serious medical problems that require acute care are not admitted. Patients at Freedom House receive medical or social detoxification depending on symptom severity. A CIWA-Ar driven protocol using lorazepam has been utilized there. The average length of stay at Freedom House is 4 days. This population represents treatment seeking individuals who are more likely to have higher levels of physical dependence than our traditional trial population recruited by advertising. This is a very important point as this population may be the one that responds best to baclofen (see Leggio et al, 2010).

Individuals at Freedom House will be screened in person whereas those who respond to advertisements will have a preliminary telephone screening conducted by the study coordinator. Individuals who appear eligible, as determined by the investigative team, will be scheduled to come to the UNC Hospitals for more comprehensive screening. Prior to full screening, individuals will read and sign the informed consent and be given a copy for their records. A breathalyzer test using an Alco-Sensor III breathalyzer machine (Intoximeters, Inc., St. Louis, MO) will be administered (must be 0.00 gms/dl to give informed consent). Height and weight will be measured and BMI calculated. Medical personnel will conduct a medical history and examination including a neurological examination. Over-the-counter and prescription medication use will be recorded. The Fagerstrom Test for Nicotine Dependence (Heatherton et al, 1991) will be administered to smokers. Laboratory evaluations will include complete blood count (CBC) with differential; chemistries including bilirubin, AST, ALT, Alkaline Phosphatase, GGT, sodium, potassium, chloride, blood urea nitrogen, creatinine, and glucose; and urinalysis and urine toxicology. A carbohydrate deficient transferrin (CDT) sample will be drawn. CDT samples will be stored frozen and then shipped in batches of 40 to Dr. Ray Anton’s lab in Charleston, South Carolina for assay. With subjects’ permission a sample of blood will also be obtained to archive for possible future genetic analyses. Women will be given a urine pregnancy test (Ub-HCG) at screening and at weeks 4, 8, 12 and 16. Trained interviewers will conduct the psychiatric screening interview using the M.I.N.I. (Sheehan et al., 1999) and the SCID Substance Use Disorders Module to establish DSM-IV criteria for alcohol dependence and to assess for other substance use disorders (First, 2002). The study coordinator will conduct the pretreatment 90-day Timeline Followback interview (TLFB, Sobell et al, 1988) and potential participants will complete the Spielberger State/Trait Anxiety Inventory (SSTAI) (Spielberger et al (1969)), the Drinker’s Inventory of Consequences (DrInC, Miller et al, 1995) the FHAM to assess family history of alcohol problems (Rice et al, 1995), the University of Rhode Island Change Assessment Scale (URICA, McConnaughy et al, 1989) to assess motivation level, the Penn Alcohol Craving Scale (PACS, Flannery et al 1999) to assess craving, and the Insomnia Severity Index (Bastien et al, 2001) to assess sleep problems. Additionally, potential participants will fill out a short evaluation of their treatment goals. This visit should take about 3 hours.

**Randomization**

A minimization random assignment procedure will be used to match subjects on the key moderator (DDD).(< 14 DDD or ≥14 DDD for men and <10 DDD or ≥10 DDD for women. This minimization random strategy for assignment to condition was developed specifically for research studies where the number of mmatching criteria is large for the number of subjects in a study (White et al,1978). In contrast to stratified randomization, which aims for equal numbers of subjects in each treatment for every possible combination of the prognostic variables, the minimization method restricts its aim to equalizing treatment numbers at the different levels of each variable taken separately. Thus, with minimization, there is not the problem that there are more combinations of variables than planned participants. Minimization is particularly superior to stratified randomization when the number of strata is large in comparison with the number of subjects (i.e., not enough subjects to fill every cell). The data analysis team has used this procedure in previous clinical trials and have found no significant differences on the matching criteria in any study (Linehan et al. 2008).

**Initial Treatment Visit**

Individuals meeting all inclusion/exclusion criteria will be informed and scheduled for the initial treatment visit within 10 days. Eligible individuals will not be required to abstain from drinking alcohol prior to randomization though those from Freedom House will be post-detoxification and may be alcohol free. The study coordinator will administer a breathalyzer test (BAC must be ≤0.04 gms/d), conduct the TLFB interview, and ask participants to complete the PACS and the SSTAI. The study physician will evaluate withdrawal symptoms using the CIWA-Ar and note the use of medications. A 1-week blister pack of baclofen/placebo with written instructions will be dispensed from the Investigational Drug Services according to the randomization block along with a 1-week back-up blister pack in case of delayed appointments or lost doses. Participants also will be given a calendar style diary to track pill taking, drinking, and any side effects and a Baclofen Instruction Sheet along with a wallet card detailing potential problems should the individual require emergency medical treatment. Finally, participants will receive Medical Management from a trained clinician. This visit should be completed in about 1.25–1.5 hours.

It is important to uptitrate and downtitrate the dose of baclofen to minimize side-effects. We will parallel the titration schedule used by Franklin et al (2009) in their study of 20 mg of baclofen given four times per day. In the latter study, baclofen was well tolerated without significant differences between baclofen and placebo groups--for the chief side-effect of sedation, the baclofen group had 20 mild, 4 moderate and 0 severe reports of sedation compared to 4 reports of mild sedation and 0 reports of either moderate or severe sedation with placebo (Franklin et al, 2009). Our titration schedule for the 90 mg/d dose is shown in Table 1. The 30 mg/d arm will reach 30 mg/d at day 3 and titrate down starting at day 101.

**Table 1:  Baclofen Titration and Dosing Schedule**

| Day | Morning Dose | Afternoon Dose | Bedtime Dose | Total Daily Dose |
| --- | --- | --- | --- | --- |
| 1-2 | placebo | 10 mg | 10 mg | 20 mg |
| 3-4 | 10 mg | 10 mg | 10 mg | 30 mg |
| 5-6 | 10 mg | 10 mg | 20 mg | 40 mg |
| 7-9 | 20 mg | 20 mg | 20 mg | 60 mg |
| 10-11 | 20 mg | 20 mg | 30 mg | 70 mg |
| 12-94 | 30 mg | 30 mg | 30 mg | 90 mg |
| 95-97 | 20 mg | 20 mg | 30 mg | 70 mg |
| 98-100 | 10 mg | 20 mg | 20 mg | 50 mg |
| 101-103 | placebo | 10 mg | 20 mg | 30 mg |
| 104-106 | placebo | placebo | 10 mg | 10 mg |
| 107 | placebo | placebo | placebo | 0 mg |

**Subsequent Treatment Visits**

At study week 1, participants will receive a 2-week supply of baclofen or placebo. After that, baclofen or placebo will be dispensed at study weeks 3, 6, 8, 10, 12 and 14. The pill taking, drinking, and side effects diaries will be collected and new diaries distributed at each visit. Medical monitoring will be conducted by study physicians and will consist of review of vital signs, concomitant medication use, and general inquiries into side effects. At each visit, participants will also be given a breathalyzer test (must be < 0.04 gms/dl), TLFB interview, and complete the PACS. Medication management sessions will be conducted at each visit. At weeks 4 and 10 blood will be drawn for evaluation of liver functioning and glucose. At weeks 4, 8, and 12 urine pregnancy testing will be conducted. At week 16 or early termination, a physical will be conducted along with CBC, chemistries, and urinalysis. Participants will be encouraged to attend Alcoholics Anonymous (AA) during treatment

**Recruitment and Retention**

It is anticipated that 120 participants can be recruited within 48 months based on prior rates of enrollment for alcohol studies conducted by the proposed study team and the awareness that the current proposal will study individuals over a longer period of time and needs to recruit a minimum number of individuals with heavy drinking and with high anxiety. In our recently completed placebo study of baclofen we were able to recruit 80 subjects over 19 months with 75% trial retention. Retention is an important objective of the trial with our target being a minimum of 75% of subjects completing the trial. Medical Management and the regular meetings with our research coordinator during study visits have proven useful in encouraging retention and compliance with medication and we have averaged 75-85% retention for most trials.

**Medical Management Intervention**

The psychosocial treatment for the proposed study will be Medical Management (MM) (Pettinati et al, 2005)). Dr. Kampov will be primarily responsible for MM with Dr. Garbutt providing back-up. Dr. Kampov and Dr. Garbutt have been trained and certified in MM by Dr. William Dundon of the University of Pennsylvania. MM is designed as a means for physicians and other health care providers to encourage individuals with AD to make progress towards their drinking goals of reduction or abstinence and to encourage compliance with medication. MM does not provide intense counseling methods for reducing drinking or dealing with relapse triggers which is thought to be an advantage for medication trials as MM does not overpower a possible medication effect.

**Baclofen Blood Level Analyses**

Baclofen (R,S-4-amino-3-p-chlorophenylbutyric acid) is marketed as a racemic mixture with the R-isomer providing essentially all the activity (see Smith, 1984). Baclofen is completely absorbed after an oral dose with much of the elimination via renal excretion (Faigle et. al, 1980). Because of the potential for active intestinal absorption that may be more important at higher doses employed in this study, as well as efforts to develop prodrugs and sustained release products of R-baclofen (Lal R et al,. 2009), it is important that the disposition of R-baclofen be examined in this efficacy study in heavy drinkers.

Baclofen trough plasma levels will be measured at week 4 at 8 hours after a dose in all participants. Trough levels will be one measure of compliance and can help to determine if some participants have unusually high exposures that could be associated with adverse events. Exploration of a relationship between trough levels and changes in drinking behavior and anxiety will be conducted as a secondary analysis.

**Enantiospecific assay of baclofen in plasma and urine.**

A wide range of assays have been employed for baclofen analysis, with a summary of enantiospecific methods provided by Goda et al. (2004). Though Goda and others have employed chiral columns or chiral mobile phases to resolve R- and S-baclofen, the indirect approach will be employed here due to its use of robust C18 columns and reduced cost. Bhushan and Kumar (2008) demonstrated that Marfey's reagent, often used for d,l-amino acid resolution allows easy separation of baclofen enanantiomers on reversed phase LC after derivatization. Baclofen will be extracted from plasma (0.10 ml) or urine (0.10 ml) by SPE (Spahn 1988), dried thoroughly, then derivatized with Marfey's reagent (1-fluoro-2,4-dinitrophenyl-5-L-alanine amide, available from Pierce/ThermoFisher) as described previously (Bhushan and Kumar, 2008), then the sample injected on LC (C18). The use of Marfey's reagent permits detection at 340 nm using UV absorbance which should be sufficient for the analysis of baclofen in plasma or urine since peak concentrations in plasma often are in the ?g/ml range. Should additional sensitivity or selectivity be needed, LC-ESI-MS/MS with an AB Sciex 3000 mass spectrometer is available for use. Validation of the LC method for the analysis of baclofen enantiomers will be done following rigorous methods as outlined within FDA guidances (FDA, 2001)

**Post-Treatment Follow-up**

One month following termination or treatment completion, the study coordinator will call each participant, conduct a TLFB interview, and inquire about general health and adverse events.

Subject participation should average around 21 weeks including screening, randomization, the active medication phase and the one month follow-up. Variations will occur for individual subjects depending upon their schedule, missed visits, etc.

# Statistical Plan

## Sample Size Determination

Our power calculations are based on the summary scores over the 12 weeks of treatment (%HDD and %ABST). Under our modified design, we must consider our 3 group design. We use Cohen's (1988) effect size definition for standardized difference for our continuous outcomes, coupled with the work of Addolorato et al. (2011) as guidelines for our power estimates and anticipated effect sizes. The Addolorato et al. (2011), similar to our design consists of 2 medication groups and a PBO reference reports contrast of incidence rates between the medication arms and PBO. Thresholds of the size of the contrasts, for comparability to the Cohen guidelines, are as defined by Rosenthal (1996) who parallels Cohen's (1988) terminology of small, medium, and larger effects for qualitative indexes such as Odds ratios and Incidence rates. Addolorato et al. (2011) saw a small to medium effect size for the contrast between PBO and 30mg. Similarly they saw a medium to large effect for the contrast between PBO and 60mg. Replicating a small effect size(Cohen's d=0.2 for our outcomes) for the contrast between PBO and 30mg coupled with a large effect size (Cohen's d=0.8 for our outcomes) for the contrast between PBO and 90mg, provides 82.2% power with 120 participants (40/arm) in testing the overall baclofen effect as well as 84.7% power to contrast 30 mg to 90 mg under the assumed effect sizes. We assume seeing a large effect size for the PBO-90mg is reasonable due to the medium-large effect seen by Addolorato et al. (2011) 60mg-PBO contrast. Interpreting the effect size for our outcomes, the PBO-overall medication contrast corresponds to a difference of 12% in %HD/%ABS rate between PBO and pooled medication sample over the 12 week period under an arc-sine transformation of the observed individual rates. The effect sizes for the contrast between medication arms corresponds to a difference of 19% in %HD/%ABS rates. We experienced 16.2% attrition in Garbutt et al (2010); therefore, a slightly larger effect size would be required to provide sufficient power for the overall BAC effect. Assuming an average effect size of 0.55, corresponds to a difference of 13% in %HDD/%ABS rate between BAC and PBO, we will have power of 82.0% to detect a statistically significant baclofen effect while accounting for attrition and 78.1% power to detect a significant difference between medication levels at the 0.6 effect size accounting for attrition.

For Aim 2, our main interest is the moderation effect of pre-treatment drinking rates on the BAC effect during treatment, for which we have some evidence as indicated in our Figure 1. Power calculations of a significant DDD x treatment interaction were implemented through SAS power procedure PROC GLMPOWER. Power derivations depended on an expected effect size, where based on the Addolorato et al. (2002) finding in a sample consisting of more DDD pre-treatment, we expect a large effect for BAC compared to PBO for those(Cohen’s d > 1), whereas Garbutt et al. (2010) in a sample consisting of lower DDD pre-treatment, found no difference (Cohen’s d=0). The pattern of this behavior is consistent with Figure 1 where more separation between BAC and PBO is evident in those with higher pre-treatment DDD. To detect this interactive behavior in our proposed design, where we assume an effect size of 1.2 corresponding to a difference of 28% in %HDD/%ABS rates in the Heavy Drinking group, and an effect size of 0 within the non-Heavy drinking group, the study will have 86.7% power to detect a significant interaction between Heavy drinking and BAC assignment. Accounting for 16.2% attrition provides 80.2% power to detect the same effect. Power calculations for the moderation assume 40 per treatment arm, with pre-treatment heavy drinking balanced per arm (20 low and 20 high).

For Aim 3, power is comparable to Aim 1, where we will have 82.2% power to detect a BAC effect f or anxiety for an effect size of 0.5 and 82.0% power to detect an effect size of 0.55 while accounting for 16.2% attrition.

## Statistical Methods

*.*

**Specific Aim 1. To test the hypothesis that baclofen, 30 mg/d and 90 mg/d, is well tolerated and superior to placebo in enhancing abstinence and reducing heavy drinking days in alcohol dependence and that 90 mg/d is superior to 30 mg/d.**

Summary measures of the proportion of abstinence days/heavy drinking days will be derived from the TLFB for the entire medication period. We will derive weekly drinking measures from TLFB as well to provide information on the impact of time on usage. Descriptive statistics and exploratory graphing will be used to assess the distribution of the data and the need for transformations. In the case of %ABS/%HDD, the arc-sine transformation is quite commonly used.

Primary Efficacy Analysis: For the Summary measure, we will fit a general linear model on the outcome variable, consisting of main effect for treatment status. Goodness-of-fit, will be analyzed for outliers and influential observations. While these summary measure approaches are simple to implement and commonly used, information concerning the effect of time on the treatment effect is ignored. To understand the impact of time, we will proceed with two additional analytical approaches based on the available data: repeated measures models and time-to-event models. From the weekly usage measures we will fit a mixed effects model consisting of main effect for treatment status. This repeated measures approach provides more information about the effect of treatment over time and therefore, more power compared to summary analyses above. We will also include the interaction of time and treatment. The interaction of time and treatment will answer the question as to whether the treatment effect is consistent over the entire treatment period or varies over the weeks. The mixed effects framework offers flexibility for the outcomes, e.g. any usage versus none, or count outcome data, e.g. number of abstinent days (Wolfinger and O’Connell, 1993). Appropriate covariance structure will be determined based on the AIC (Akakie Information Criteria) and BIC (Bayesian Information Criteria). Cox regression will be used to assess baclofen effect for time outcomes, e.g. time to first use (Collett (1994). We will evaluate the baclofen effect for changes in GGT, and CDT using the mixed model approach described above. Within all framework, statistical contrasts will allow us to compare the two levels of BAC to PBO, as well as statistically contrasting the two levels of BAC. The advantage of the statistical contrasts is it allows us to use all available data to estimate the variance (variance-covariance for the repeated measures) of the outcome, while also properly distributing the degrees of freedom for the analytical test to account for the three levels of treatment.

Treatment of Missing Data: With repeated assessments, missing data/attrition is inevitable, but the key thing is that the specified contrasts are not affected due to the presence/absence of data. We will use pattern-mixture models to assess if there is bias due to drop out or missing data (Hedeker and Gibbons, 1997). To address missing data in the survival models, Dupuy and Mesbah(2004) considered jointly modeling time to event data with missing covariate data in order to assess if the missing data is ignorable or non-ignorable. These approaches will be implemented to the fitted cox model. Days in treatment may directly impact the treatment comparison for assessing differences. To control for this potential issue, we will also assess Marginal Structural Cox Regression models (Xiao et al., 2010).

Adjustment for Covariates: All modeling approaches allow for the inclusion of covariates. We will include any measures differing at baseline or important potentially confounding measures (i.e. gender, race) in our models to disentangle the intervention effect from these measures

**Specific Aim 2. To test the hypothesis that a history of heavy drinking as assessed by average drinks/drinking day in the 30 days preceding screening predicts baclofen response in alcohol dependence.**

We will include the pre-treatment HDD measure in all models above to examine its predictive merit, as well as its potential moderating impact of the baclofen effect. Moderation effect will be assessed by including the interaction of pre-treatment HDD with the treatment assignment. We will treat pre-treatment HDD as a continuous measure. We will use mean profile plots based on quartiles of HDD to assess if the continuous measure has a linear, piecewise linear, or curvilinear effect. .

**Specific Aim 3. To test the hypothesis that baclofen, 30 mg/d and 90 mg/d, is superior to placebo in reducing anxiety in alcohol dependence.**

Our analytic procedure is as described in Aim 1 using mixed effects model to account for the repeated measures over time, while contrasting the mean intervention effect as well as assessing the potenetial moderation effect of pre-treatment HDD.

# Safety and Adverse Events

## Definitions

**Unanticipated Problems Involving Risk to Subjects or Others**

Any incident, experience, or outcome that meets all of the following criteria:

- Unexpected in nature, severity, or frequency (i.e. not described in study-related documents such as the IRB-approved protocol or consent form, the investigators brochure, etc)
- Related or possibly related to participation in the research (i.e. possibly related means there is a reasonable possibility that the incident experience, or outcome may have been caused by the procedures involved in the research,
- Serious (as defined below) “***Serious” is different than “severe” as reported in the CTC criteria that applies a grade to the AE.***

**Adverse Event**

An ***adverse event*** (AE) is any symptom, sign, illness or experience that develops or worsens in severity during the course of the study. Intercurrent illnesses or injuries should be regarded as adverse events. Abnormal results of diagnostic procedures are considered to be adverse events if the abnormality:

- results in study withdrawal
- is associated with a serious adverse event
- is associated with clinical signs or symptoms
- leads to additional treatment or to further diagnostic tests
- is considered by the investigator to be of clinical significance

**Serious Adverse Event**

Adverse events are classified as serious or non-serious. A ***serious adverse event*** is any AE that is:

- fatal
- life-threatening
- requires or prolongs hospital stay
- results in persistent or significant disability or incapacity
- a congenital anomaly or birth defect
- an important medical event

Important medical events are those that may not be immediately life threatening, but are clearly of major clinical significance. They may jeopardize the subject, and may require intervention to prevent one of the other serious outcomes noted above. For example, drug overdose or abuse, a seizure that did not result in in-patient hospitalization, or intensive treatment of bronchospasm in an emergency department would typically be considered serious.

All adverse events that do not meet any of the criteria for serious should be regarded as ***non-serious adverse events***.

For the purpose of this trial the following events are expected side effects of the drug:

- Drowsiness
- Weakness
- Dizziness/lightheadedness
- Nausea/vomiting
- Headache
- Insomnia
- Lethargy/fatigue
- Frequent urination
- Constipation
- Confusion
- Hypotension
- Blurred vision
- Seizures (rare)

**Adverse Event Reporting Period**

The study period during which adverse events must be reported is normally defined as the period from the initiation of any study procedures to the end of the study treatment follow-up. For this study, the study treatment follow-up is defined as 30 days following the last administration of study treatment.

**Preexisting Condition**

A preexisting condition is one that is present at the start of the study. A preexisting condition should be recorded as an adverse event if the frequency, intensity, or the character of the condition worsens during the study period.

**General Physical Examination Findings**

At screening, any clinically significant abnormality should be recorded as a preexisting condition. At the end of the study, any new clinically significant findings/abnormalities that meet the definition of an adverse event must also be recorded and documented as an adverse event.

**Post-study Adverse Event**

All unresolved adverse events should be followed by the investigator until the events are resolved, the subject is lost to follow-up, or the adverse event is otherwise explained. At the last scheduled visit, the investigator should instruct each subject to report any subsequent event(s) that the subject, or the subject’s personal physician, believes might reasonably be related to participation in this study. The investigator should notify the study sponsor of any death or adverse event occurring at any time after a subject has discontinued or terminated study participation that may reasonably be related to this study. The sponsor should also be notified if the investigator should become aware of the development of cancer or of a congenital anomaly in a subsequently conceived offspring of a subject that has participated in this study.

**Abnormal Laboratory Values**

A clinical laboratory abnormality should be documented as an adverse event if any one of the following conditions is met:

- The laboratory abnormality is not otherwise refuted by a repeat test to confirm the abnormality
- The abnormality suggests a disease and/or organ toxicity
- The abnormality is of a degree that requires active management; e.g. change of dose, discontinuation of the drug, more frequent follow-up assessments, further diagnostic investigation, etc.

Because baclofen is principally renally excreted individuals with elevated creatinine or reduced GFR will be excluded. Additionally, creatinine and a liver profile will be monitored at 4, 8 and 12 weeks and, if creatinine is elevated or AST/ALT levels > 5 X ULN, medication will be stopped. Participants will be given a card to carry with them to inform health care personnel that the individual may be on baclofen.

**Hospitalization, Prolonged Hospitalization or Surgery**

Any adverse event that results in hospitalization or prolonged hospitalization should be documented and reported as a serious adverse event unless specifically instructed otherwise in this protocol. Any condition responsible for surgery should be documented as an adverse event if the condition meets the criteria for and adverse event.

Neither the condition, hospitalization, prolonged hospitalization, nor surgery are reported as an adverse event in the following circumstances:

- Hospitalization or prolonged hospitalization for diagnostic or elective surgical procedures for a preexisting condition. Surgery should ***not*** be reported as an outcome of an adverse event if the purpose of the surgery was elective or diagnostic and the outcome was uneventful.
- Hospitalization or prolonged hospitalization required to allow efficacy measurement for the study.
- Hospitalization or prolonged hospitalization for therapy of the target disease of the study, unless it is a worsening or increase in frequency of hospital admissions as judged by the clinical investigator.

## Recording of Adverse Events

At each contact with the subject, the investigator must seek information on adverse events by specific questioning and, as appropriate, by examination. Information on all adverse events should be recorded immediately in the source document, and also in the appropriate adverse event module of the case report form (CRF). All clearly related signs, symptoms, and abnormal diagnostic procedures results should recorded in the source document, though should be grouped under one diagnosis.

All adverse events occurring during the study period must be recorded. The clinical course of each event should be followed until resolution, stabilization, or until it has been determined that the study treatment or participation is not the cause. Serious adverse events that are still ongoing at the end of the study period must be followed up to determine the final outcome. Any serious adverse event that occurs after the study period and is considered to be possibly related to the study treatment or study participation should be recorded and reported immediately.

## Reporting of Serious Adverse Events and Unanticipated Problems

Investigators and the protocol sponsor must conform to the adverse event reporting timelines, formats and requirements of the various entities to which they are responsible, but at a minimum those events that must be reported are those that are:

- related to study participation,
- unexpected, and
- serious or involve risks to subjects or others

(see definitions, section 8.1).

If the report is supplied as a narrative, the minimum necessary information to be provided at the time of the initial report includes:

| - Study identifier - Study Center - Subject number - A description of the event - Date of onset | - Current status - Whether study treatment was discontinued - The reason why the event is classified as serious - Investigator assessment of the association between the event and study treatment |
| --- | --- |

### Investigator reporting:

For reportable deaths, the initial submission to the UNC IRB may be made by contacting the IRB Director or Associate Director. The AE/Unanticipated Problem Form is required as a follow up to the initial submission.

**Other Reportable events:**

For clinical drug trials, the following events are also reportable to the UNC IRB:

- Any adverse experience that, even without detailed analysis, represents a serious unexpected adverse event that is rare in the absence of drug exposure (such as agranulocytosis, hepatic necrosis, Stevens-Johnson syndrome).
- Any adverse event that would cause the sponsor to modify the investigators brochure, protocol or informed consent form, or would prompt other action by the IRB to assure protection of human subjects.
- Information that indicates a change to the risks or potential benefits of the research, in terms of severity or frequency. For example:
- An interim analysis indicates that participants have a lower rate of response to treatment than initially expected.
- Safety monitoring indicates that a particular side effect is more severe, or more frequent than initially expected.
- A paper is published from another study that shows that an arm of your research study is of no therapeutic value.
- Change in FDA safety labeling or withdrawal from marketing of a drug, device, or biologic used in a research protocol.
- Breach of confidentiality
- Change to the protocol taken without prior IRB review to eliminate apparent immediate hazard to a research participant.
- Incarceration of a participant when the research was not previously approved under Subpart C and the investigator believes it is in the best interest of the subject to remain on the study.
- Complaint of a participant when the complaint indicates unexpected risks or the complaint cannot be resolved by the research team.
- Protocol violation (meaning an accidental or unintentional deviation from the IRB approved protocol) that in the opinion of the investigator placed one or more participants at increased risk, or affects the rights or welfare of subjects.

### Sponsor reporting: Notifying the FDA

The study sponsor is required to report certain study events in an expedited fashion to the FDA. These written notifications of adverse events are referred to as IND safety reports. The following describes the safety reporting requirements by timeline for reporting and associated type of event:

- ***Within 7 calendar days***

Any study event that is:

- associated with the use of the study drug
- unexpected,
- fatal or life-threatening, and
- ***Within 15 calendar days***

Any study event that is:

- associated with the use of the study drug,
- unexpected, and
- serious, but not fatal or life-threatening

-or-

- a previous adverse event that was not initially deemed reportable but is later found to fit the criteria for reporting (reporting within 15 calendar days from when event was deemed reportable).

Any finding from tests in laboratory animals that:

- suggest a significant risk for human subjects including reports of mutagenicity, teratogenicity, or carcinogenicity.

**Additional reporting requirements**

Sponsors are also required to identify in IND safety reports all previous reports concerning similar adverse events and to analyze the significance of the current event in light of the previous reports.

**Reporting Process**

Adverse events will be submitted on FDA Form 3500A or in a narrative format, along with FDA form 1571. If supplied as in a narrative format, the minimum information to be supplied is noted above at the beginning of section 8.3.

.

## Unblinding Procedures

The randomization schedule will be given to IDS personnel; investigators will not have access to this information. If a medical or other emergency necessitates breaking the blind, IDS personnel will provide the identity of the medication to the PI or another medical study team member. This will be recorded in the subject’s study record.

## 8.5 Stopping Rules

The Data Safety Monitoring Board (DSMB) will provide a summary letter to the PI and to the IRB regarding any safety concerns and a recommendation as to whether the study should be stopped or modified for safety reasons. No a priori stopping rules will be established for this Phase II trial.

A subject who experiences a serious adverse event thought to be possibly or probably related to study medication will have their medication tapered and stopped. It is preferable to taper baclofen then stopping it abruptly. If a subject is having moderate side-effects the medication can be reduced and side-effects monitored. If a subject continues to have side-effects the medication may be tapered and stopped. We will make every effort to maintain subjects in the trial as long as safety is adhered to. If a subject does not comply with medication instructions we may need to discontinue medication for safety purposes. We will follow subjects off medication to collect outcome data such as drinking behavior.

## Medical Monitoring

The study PI, Dr. Garbutt, will be responsible for monitoring participant safety during the trial and will be assisted by other study physicians and research coordinators. Dr. Garbutt has extensive experience in clinical trials for alcohol dependence including previous experience with baclofen. Serious adverse events will be reported to the IRB, to the DSMB and to the NIAAA project officer within 24 hours. An annual report documenting all adverse events will be submitted to the NIAAA project officer.

Participants will be contacted by telephone 4 weeks after the final trial visit to ascertain drinking behavior and any physical or psychiatric problems. Individuals with clinically significant problems will be referred for appropriate psychiatric or medical treatment.

No alcohol will be administered to any individual as part of this research project.

### Data and Safety Monitoring Board

Data Safety Monitoring Board (DSMB): A DSMB will be established given that the trial will represent the first placebo-controlled trial of high dose baclofen for alcohol dependence. Dr. Cort Pedersen, Professor of Psychiatry at UNC-Chapel Hill, will serve as the Chair of the DSMB. Dr. Pedersen is an experienced clinician who has worked with patients with substance use disorders for many years. Dr. Pedersen is also an active clinical researcher and has familiarity with clinical trials. Dr. George Kenna, Assistant Professor of Psychiatry and Human Behavior at Brown University, will serve on the DSMB. Dr. Kenna is a pharmaceutical scientist with experience in clinical trials who has knowledge of the use of baclofen in alcohol dependence. Dr. Bob Gallop, Associate Professor of Statistics at West Chester University and the statistician for the trial, will serve on the DSMB. Dr. Gallop will have knowledge of how to interpret the dataset and will be familiar with the randomization schedule. Dr. Gallop will provide unblinded summaries of the trial to the DSMB.

The DSMB will meet once 20 participants have been randomized and then will meet at six month intervals. Meetings will be by teleconference. The DSMB will provide a summary letter to the PI and to the IRB regarding any safety concerns and a recommendation as to whether the study should be stopped or modified for safety reasons. No a priori stopping rules will be established for this Phase II trial.

# Data Handling and Record Keeping

## Confidentiality

Information about study subjects will be kept confidential and managed according to the requirements of the Health Insurance Portability and Accountability Act of 1996 (HIPAA). Those regulations require a signed subject authorization informing the subject of the following:

- What protected health information (PHI) will be collected from subjects in this study
- Who will have access to that information and why
- Who will use or disclose that information
- The rights of a research subject to revoke their authorization for use of their PHI.

In the event that a subject revokes authorization to collect or use PHI, the investigator, by regulation, retains the ability to use all information collected prior to the revocation of subject authorization. For subjects that have revoked authorization to collect or use PHI, attempts should be made to obtain permission to collect at least vital status (i.e. that the subject is alive) at the end of their scheduled study period.

## Source Documents

Source data is all information, original records of clinical findings, observations, or other activities in a clinical trial necessary for the reconstruction and evaluation of the trial. Source data are contained in source documents. Examples of these original documents, and data records include: hospital records, clinical and office charts, laboratory notes, memoranda, subjects’ diaries or evaluation checklists, pharmacy dispensing records, recorded data from automated instruments, copies or transcriptions certified after verification as being accurate and complete, microfiches, photographic negatives, microfilm or magnetic media, x-rays, subject files, and records kept at the pharmacy, at the laboratories, and at medico-technical departments involved in the clinical trial.

## Data Collection

Subjects will provide information on alcohol use patterns and symptoms. Mental health symptoms, physical symptoms and general demographic data will be obtained via open-ended interviews and structured questionnaires. Blood and urine samples will be gathered. All data will be entered into case-report forms and then into a secure electronic data base. Subjects will not be identified by name or unique identifying information in the case-report forms or in the electronic data base. A linkage file will be maintained using a code and this will be kept in a secure file cabinet and secure office. The linkage file will only be available to the PI, Co-PIs, and research coordinators.

The Database will be configured in Microsoft Access. Discrepancies will be examined and additional data checks completed as indicated. A closed and password-protected data entry system will be used and only certified study personnel will be able to enter or edit data. Data and user stamping are used to create an audit trail. Range checks, review screens, and error trapping routines are built into the system as quality control procedures. Hardcopy data summaries will be available to the investigators for data analyses. As a final check on data integrity, the investigators and study coordinator will check these outputs against the raw data and resolve any discrepancies. A back up of all archival data will be saved on DVD on a monthly basis.

## Records Retention

It is the investigator’s responsibility to retain study essential documents for at least 2 years after the last approval of a marketing application in their country and until there are no pending or contemplated marketing applications in their country or at least 2 years have elapsed since the formal discontinuation of clinical development of the investigational product. These documents should be retained for a longer period if required by an agreement with the sponsor. In such an instance, it is the responsibility of the sponsor to inform the investigator/institution as to when these documents no longer need to be retained.

# Study Monitoring, Auditing, and Inspecting

## Study Monitoring Plan

The investigator will allocate adequate time for such monitoring activities. The Investigator will also ensure that the monitor or other compliance or quality assurance reviewer is given access to all the above noted study-related documents and study related facilities (e.g. pharmacy, diagnostic laboratory, etc.), and has adequate space to conduct the monitoring visit.

We will follow our standard protocol for monitoring of clinical trials. First, subjects are screened to exclude those who might have an increased risk associated with study participation, e.g. renal failure, diabetes mellitus, pregnancy. Pregnancy will be initially screened using a blood test with increased sensitivity compared to urine tests. Second, subjects are monitored on a regular basis with visits at post-randomization weeks 1, 2, 3, 4, 6, 8, 10, 12, 14 and 16. At these visits vital signs are taken and subjects are asked about any side-effects or problems that they are having. At weeks 4, 8, 12 and at termination a liver profile and creatinine level are examined. Participants with elevated creatinine or AST/ALT levels > 5X ULN or a bilirubin > 1.5 ULN will have their medication tapered and stopped and will be monitored by study physicians. In these instances another liver profile and creatinine will be drawn within 2-4 weeks.

Participants will be contacted by telephone 4 weeks after the final trial visit to ascertain drinking behavior and any physical or psychiatric problems. Individuals with clinically significant problems will be referred for appropriate psychiatric or medical treatment.

## Auditing and Inspecting

The investigator will permit study-related monitoring, audits, and inspections by the IRB, government regulatory bodies, and University compliance and quality assurance groups of all study related documents (e.g. source documents, regulatory documents, data collection instruments, study data etc.). The investigator will ensure the capability for inspections of applicable study-related facilities (e.g. pharmacy, diagnostic laboratory, etc.).

Participation as an investigator in this study implies acceptance of potential inspection by government regulatory authorities and applicable University compliance and quality assurance offices.

# Ethical Considerations

This study is to be conducted according to US and international standards of Good Clinical Practice (FDA Title 21 part 312 and International Conference on Harmonization guidelines), applicable government regulations and Institutional research policies and procedures.

This protocol and any amendments will be submitted to a properly constituted independent Ethics Committee (EC) or Institutional Review Board (IRB), in agreement with local legal prescriptions, for formal approval of the study conduct. The decision of the EC/IRB concerning the conduct of the study will be made in writing to the investigator and a copy of this decision will be provided to the sponsor before commencement of this study. The investigator should provide a list of EC/IRB members and their affiliate to the sponsor.

All subjects for this study will be provided a consent form describing this study and providing sufficient information for subjects to make an informed decision about their participation in this study. This consent form will be submitted with the protocol for review and approval by the EC/IRB for the study. The formal consent of a subject, using the EC/IRB-approved consent form, must be obtained before that subject undergoes any study procedure. The consent form must be signed by the subject or legally acceptable surrogate, and the investigator-designated research professional obtaining the consent.

# Study Finances

## Funding Source

This study is funded, in part, by a grant from the NIH.

## Conflict of Interest

Any investigator who has a conflict of interest with this study (patent ownership, royalties, or financial gain greater than the minimum allowable by their institution, etc.) must have the conflict reviewed by a properly constituted Conflict of Interest Committee with a Committee-sanctioned conflict management plan that has been reviewed and approved by the study sponsor prior to participation in this study. All University of UNC investigators will follow the University conflict of interest policy.

## Subject Stipends or Payments

Subjects will receive $10 for the randomization visit and each of the post-randomization visits. They will receive an additional $50 for completing the study. If they complete all visits and complete the study they will receive $160. If subject drops out before completing the study they will not receive the $50 completion bonus. Subjects will also receive parking passes as needed.

# Publication Plan

Neither the complete nor any part of the results of the study carried out under this protocol, nor any of the information provided by the sponsor for the purposes of performing the study, will be published or passed on to any third party without the consent of the study sponsor. Any investigator involved with this study is obligated to provide the sponsor with complete test results and all data derived from the study.

# References

1. Addolorato, G., Caputo, F., Capristo, E., Colombo, G., Gessa, G.L., & Gasbarrini, G. (2000). Ability of baclofen in reducing alcohol craving and intake: II-preliminary clinical evidence. Alcohol: Clin Exp Res 24:67-71.
2. Addolorato, G., Caputo, F., Capristo, E., et al. (2002). Baclofen efficacy in reducing alcohol craving and intake:A preliminary double-blind randomized controlled study. Alcohol Alcohol 37: 504-508.
3. Addolorato G, Leggio L, Ferrulli A, Cardone S, Vonghia L, Mirijello A, Abenavoli L, D'Angelo C, Caputo F, Zambon A, Haber PS, Gasbarrini G (2007). Effectiveness and safety of baclofen for maintenance of alcohol abstinence in alcohol-dependent patients with liver cirrhosis: randomised, double-blind controlled study. Lancet. 370:1915-1922
4. Addolorato G, Leggio L, Ferrulli A, Cardone S, Bedogni G, Caputo F, Gasbarrini G, Landolfi R; Baclofen Study Group (2011). Dose-response effect of baclofen in reducing daily alcohol intake in alcohol dependence: secondary analysis of a randomized, double-blind, placebo-controlled trial. Alcohol Alcohol. 46:312-17
5. Ameisen O (2005). Complete and prolonged suppression of symptoms and consequences of alcoholdependence using high-dose baclofen: a self-case report of a physician. Alcohol Alcohol 40:147-50.
6. Anton RF, O'Malley SS, Ciraulo DA, Cisler RA, Couper D, Donovan DM, Gastfriend DR, Hosking JD, Johnson BA, LoCastro JS, Longabaugh R, Mason BJ, Mattson ME, Miller WR, Pettinati HM, Randall CL, Swift R, Weiss RD, Williams LD, Zweben A; COMBINE Study Research Group (2006). Combined pharmacotherapies and behavioral interventions for alcohol dependence: the COMBINE study: a randomized controlled trial. JAMA.295:2003-17.
7. Bastien CH, Vallières A, Morin CM (2001). Validation of the Insomnia Severity Index as an outcome measure for insomnia research. Sleep Med 2:297-307.
8. Bhushan R and Kumar V (2008). Indirect resolution of baclofen enantiomers from pharmaceutical dosage for by reversed-phase liquid chromatography after derivatization with Marfey's reagent and its structural variants. Biomed Chromatogr 22:906-911.
9. Bouchery EE, Harwood HJ, Sacks JJ, Simon CJ, Brewer RD (2011). Economic Costs of Excessive Alcohol Consumption in the U.S 2006. Am J Prev Med 41:516–524.
10. Breslow MF, Fankhauser MP, Potter RL, Meredith KE, Misiaszek J, Hope DG Jr (1989). Role of gammaaminobutyric acid in antipanic drug efficacy. Am J Psychiatry. 146:353-6.
11. Bucknam W (2007). Suppression of symptoms of alcohol dependence and craving using high-dose baclofen. Alcohol Alcohol. 42:158-60.
12. Cohen J (1988). Statistical power analysis for the behavioral sciences. 2nd ed. Hillsdale, NJ: Lawrence Erlbaum Associates.
13. Collett D (1994). Modeling Survival Data in Medical Research New York, NY: Chapman and Hall. Cott J, Carlsson A, Engel J, Lindqvist M (1976). Suppression of ethanol-induced locomotor stimulation by GABA-like drugs. Naunyn Schmiedebergs Arch Pharmacol. 295(3):203-9.
14. Cryan JF, Kaupmann K (2005). Don't worry 'B' happy!: a role for GABA(B) receptors in anxiety and depression. Trends Pharmacol Sci. 26(1):36-43
15. Drake RG, Davis LL, Cates ME, Jewell ME, Ambrose SM, Lowe JS (2003). Baclofen treatment for chronic posttraumatic stress disorder. Ann Pharmacother 37:1177-81.
16. Dupuy JF,, Mesbah M (2004). Estimation of the asymptotic variance of semiparametric maximum likelihood estimators in the Cox model with missing time-dependent covariate. Communications in Statistics - Theory and Methods 33: 1385-401.
17. Evans SM, Bisaga A (2009). Acute interaction of baclofen in combination with alcohol in heavy social drinkers. Alcohol Clin Exp Res 33:19-30.
18. Faigle JW, Keberle H, and Degen PH (1980) Chemistry and pharmacokinetics of baclofen, in Spasticity:Disordered Motor Control (Feldman RG, Young RR, Koella WP, eds) pp 461-475, Ciba-Giegy Corp/Symposium Specialists, Miami, Fl.
19. File SE, Zharkovsky A, Gulati K (1991). Effects of baclofen and nitrendipine on ethanol withdrawal responses in the rat. Neuropharmacology. 30:183-90. References Cited Page 57 Principal Investigator/Program Director (Last, first, middle): Garbutt, James, C
20. First, Michael B., Spitzer, Robert L, Gibbon Miriam, and Williams, Janet B.W (2002). Structured Clinical Interview for DSM-IV-TR Axis I Disorders, Research Version, Patient Edition. (SCID-I/P) New York: Biometrics Research, New York State Psychiatric Institute. FDA Guidance (2001): http://www.fda.gov/downloads/Drugs/GuidanceComplianceRegulatoryInformation/Guidances/UCM070107.pdf
21. Flannery BA, Garbutt JC, Cody MW, Renn W, Grace K, Osborne M, Crosby K, Morreale M, Trivette A (2004). Baclofen for alcohol dependence: a preliminary open-label study. Alcohol Clin Exp Res 28:1517-23.
22. Flannery, B.A., Volpicelli, J.R., & Pettinati, H.M. (1999). Psychometric properties of the Penn Alcohol Craving Scale. Alcohol Clin Exp Res 23:1289-1295.
23. Franklin TR, Harper D, Kampman K, Kildea-McCrea S, Jens W, Lynch KG, O'Brien CP, Childress AR. The GABA B agonist baclofen reduces cigarette consumption in a preliminary double-blind placebo-controlled smoking reduction study. Drug Alcohol Depend. 2009 103:30-6.
24. Gaillard JM. Comparison of two muscle relaxant drugs on human sleep: diazepam and parachlorophenylgaba. Acta Psychiatr Belg 77:410–425.
25. Garbutt JC, Kampov-Polevoy AB, Gallop R, Kalka-Juhl L, Flannery BA (2010). Efficacy and safety of baclofen for alcohol dependence: a randomized, double-blind, placebo-controlled trial. Alcohol Clin Exp Res 34:1849- 57.
26. Goda R, Murayama N, Fujimaki Y and Sudo K (2004). Simple and sensitive liquid chromatography-tandem mass spectrometry method for determination of the S(+)- and R(-)-enantiomers of baclofen in human plasma. B J Chromatogr 801:257-264.
27. Grant BF, Stinson FS; Deborah A. Dawson, PhD; S. Patricia Chou, PhD;Mary C. Dufour, MD, MPH; Wilson Compton, MD; Roger P. Pickering, MS; Kenneth Kaplan, BS (2004). Prevalence and Co-occurrence of Substance UseDisorders and Independent Mood and Anxiety Disorders. Results From the National Epidemiologic Survey on Alcohol and Related Conditions. Arch Gen Psychiatry 61:807-816 Heatherton TF, Kozlowski LT, Frecker RC, Fagerström KO (1991). The
28. Fagerström test for nicotine dependence: a revision of the Fagerström Tolerance Questionnaire. Br J Addict 86:1119-27
29. Hedeker, D. & Gibbons, R.D. (1997). Application of random-effects pattern-mixture models for missing data in longitudinal studies. Psychological Methods,2, 64-78.
30. Holstein SE, Dobbs L, Phillips TJ (2009). Attenuation of the stimulant response to ethanol is associated with enhanced ataxia for a GABAA, but not a GABAB, receptor agonist. Alcohol Clin Exp Res 33:108-20.
31. Kahn R, Biswas K, Childress AR, Shoptaw S, Fudala PJ, Gorgon L, Montoya I, Collins J, McSherry F, Li SH, Chiang N, Alathari H, Watson D, Liberto J, Beresford T, Stock C, Wallace C, Gruber V, Elkashef A (2009). Multi-center trial of baclofen for abstinence initiation in severe cocaine-dependent individuals. Drug Alcohol Depend. 103:59-64.
32. Knapp DJ, Overstreet DH, Breese GR (2007). Baclofen blocks expression and sensitization of anxiety-like behavior in an animal model of repeated stress and ethanol withdrawal. Alcohol Clin Exp Res 31:582-95.
33. Kranzler HR, Van Kirk J (2001). Efficacy of naltrexone and acamprosate for alcoholism treatment: a metaanalysis. Alcohol Clin Exp Res 25:1335-41.

34. Krupitsky, E.M., Burakov, A.M., Ivano, V.B., Krandashova, I.P., Lapin, A., Ja Grinenko, A., & Borodkin, Y.S. (1993). Baclofen administration for the treatment of affective disorders in alcoholic patients. Drug Alcohol Depend 33:157-163.

35. Kushner MG, Abrams K, Borchardt C (2000). The relationship between anxiety disorders and alcohol use disorders: a review of major perspectives and findings. Clin Psychol Rev 20:149-71.

36. Lal R, Sukbuntherng J, Tai EHL, et al. (2009). Arbaclofen placarbil, a novel R-baclofen prodrug: Improved absorption, distribution, metabolism and elimination properties compared with R-baclofen. J Pharmacol Exp Ther 330:911-921.

37. Leggio L, Garbutt JC, Addolorato G (2010). Effectiveness and Safety of Baclofen in the Treatment of Alcohol Dependent Patients. CNS & Neurological Disorders - Drug Targets 2010:33-44

38. Leung NY, Whyte IM, Isbister GK (2006). Baclofen overdose: defining the spectrum of toxicity. Emerg Med Australas 18:77-82. References Cited Page 58 Principal Investigator/Program Director (Last, first, middle): Garbutt, James, C

39. Linehan, M.M., McDavid, J., Brown, M.Z., Sayrs, J.H.R., and Gallop, R.J. (2008). Olanzapine plus dialectical behavior therapy for irritable women meeting criteria for borderline personality disorder: a double blind, placebo-controlled pilot study. J Clin Psychiatry 69:999-1005.

40. Lyon JE, Khan RA, Gessert CE, Larson PM, Renier CM (2011). Treating alcohol withdrawal with oral baclofen: A randomized, double-blind, placebo-controlled trial. J Hosp Medicine 6:469-474

41. Maccioni P, Colombo G (2009). Role of the GABA(B) receptor in alcohol-seeking and drinking behavior. Alcohol 43:555-8. Maccioni P, Carai MA, Kaupmann K, Guery S, Froestl W, Leite-Morris KA, Gessa GL, Colombo G (2009). Reduction of alcohol's reinforcing and motivational properties by the positive allosteric modulator of the GABA(B) receptor, BHF177, in alcohol-preferring rats. Alcohol Clin Exp Res.33:1749-56

42. Mason BJ, Goodman AM, Chabac S, Lehert P (2006). Effect of oral acamprosate on abstinence in patients with alcohol dependence in a double-blind, placebo-controlled trial: the role of patient motivation. J Psychiatr Res 40:383-93.

43. McConnaughy DA, DiClemente CC, Prochaska JO, Velicer WF (1989). Stages of change in psychotherapy: a follow-up report. Psychotherapy: Theory, Research and Practice 4:494-503.

44. Miller, W. R., Tonigan, J. S., & Longabaugh, R. (1995). The Drinker Inventory of Consequences (DrInC): An instrument for assessing adverse consequences of alcohol abuse. Project MATCH Monograph Series, Vol. 4. Rockville, MD: NIAAA.

45. Miller WR, Walters ST, Bennett ME (2001). How effective is alcoholism treatment in the United States? J Stud Alcohol 62:211-20.

46. Mokdad A, Marks J, Stroup D, Gerberding J (2004). Actual causes of death in the United States, 2000. JAMA 291:1238-45

47. O'Malley SS, Jaffe AJ, Chang G, Schottenfeld RS, Meyer RE, Rounsaville B (1992). Naltrexone and coping skills therapy for alcohol dependence. A controlled study. Arch Gen Psychiatry 49:881-7.

48. Pastor A, Jones DM, Currie J (2012). High-dose baclofen for treatment-resistant alcohol dependence. J Clin Psychopharmacol. Feb 23. [Epub ahead of print].

49. Pettinati HM, Weiss RD, Dundon W, Miller WR, Donovan D, Ernst DB, Rounsaville BJ (2005). A structured approach to medical management: a psychosocial intervention to support pharmacotherapy in the treatment of alcohol dependence. J Stud Alcohol Suppl 15:170-8.

50. Rice JP, Reich T, Bucholz KK, Neuman RJ, Fishman R, Rochberg N, Hesselbrock VM, Nurnberger JI Jr, Schuckit MA, Begleiter H (1995). Comparison of direct interview and family history diagnoses alcohol dependence. Alcohol Clin Exp Res 19:1018-23.

51. Rosenthal, J.A. (1996). Qualitative Descriptors of Strength of Association and Effect Size, J Social Service Res 21:37-59.

1. Sheehan, D., Janavs, J., Baker, R., Harnett-Sheehan, K., Knapp, E., & Sheenan, M. (1999). Mini International Neuropsychiatric Interview (M.I.N.I.). Tampa: University of South Florida.
2. Smith DF (1984). Stereoselectivity of spinal neurotransmission: effects of baclofen enantiomer on tail-flick reflex in rats. J Neural Transm 60:63-7.

54. Sobell, L.C., Sobell, M.B., Leo, G.L., & Cancilla, A. (1988). Reliability of a timeline followback method: Assessing normal drinkers’ reports of recent drinking and a comparative evaluation across several populations. Brit J Addictions, 83, 393-402.

55. Spahn H, Krauss D and Mutshcler E (1988). Enatiospecific high-performance liquid chromatographic (HPLC) determination of baclofen and its fluoro analog in biological material. Pharmaceut Res 5:107-112.

56. Speilberger, C.D., Gorsuch, R.L., & Lushene, R.E. (1969). The state trait anxiety inventory manual. Palo Alto: Consulting Psychologists Press.

57. Sullivan, J.T., Sykora, K., Schneiderman, J., Naranjo, C.A., & Sellers, E.M. (1989). Assessment of alcohol withdrawal: The revised Clinical Institute Withdrawal Assessment of Alcohol Scale (CIWA-Ar). British Journalof Addictions, 84, 1353-1357.

58. Volpicelli JR, Rhines KC, Rhines JS, Volpicelli LA, Alterman AI, O'Brien CP (1997). Naltrexone and alcohol dependence. Role of subject compliance. Arch Gen Psychiatry 54:737-42 References Cited Page 59 Principal Investigator/Program Director (Last, first, middle): Garbutt, James, C

59. Volpicelli JR, Alterman AI, Hayashida M, O'Brien CP (1992). Naltrexone in the treatment of alcohol dependence. Arch Gen Psychiatry 49:876-80. Wall GC, Wasiak A, Hicklin GA (2006). An initially unsuspected case of baclofen overdose. Am J Crit Care. 5:611-3.

60. White SJ, Freedman LS (1978). Allocation of patients to treatment groups in a controlled clinical study. Br J Cancer 37: 849-57.

61. Wolfinger R.D. & O'Connell N. (1993). Generalized linear mixed models: a pseudo likelihood approach. J Stat Comp Simulation 48:233-243.

62. Xiao Y, Abramawics M, Moodie EEM (2010). Accuracy of conventional and marginal structural cox model estimates: A simulation story. Int J Biostatistics 6:1-30.
